# The Breeding of Winter-Hardy Malting Barley

**DOI:** 10.3390/plants10071415

**Published:** 2021-07-11

**Authors:** Eric J. Stockinger

**Affiliations:** Ohio Agricultural Research and Development Center (OARDC), Department of Horticulture and Crop Science, The Ohio State University, Wooster, OH 44691, USA; stockinger.4@osu.edu

**Keywords:** winter-hardiness, *FROST RESISTANCE-H2*, CBF transcription factors, gene regulation, copy number variation, *Rht-1* semi-dwarfing genes, GA-GID1-DELLA module, circadian clock

## Abstract

In breeding winter malting barley, one recurring strategy is to cross a current preferred spring malting barley to a winter barley. This is because spring malting barleys have the greatest amalgamation of trait qualities desirable for malting and brewing. Spring barley breeding programs can also cycle their material through numerous generations each year—some managing even six—which greatly accelerates combining desirable alleles to generate new lines. In a winter barley breeding program, a single generation per year is the limit when the field environment is used and about two generations per year if vernalization and greenhouse facilities are used. However, crossing the current favored spring malting barley to a winter barley may have its downsides, as winter-hardiness too may be an amalgamation of desirable alleles assembled together that confers the capacity for prolonged cold temperature conditions. In this review I touch on some general criteria that give a variety the distinction of being a malting barley and some of the general trends made in the breeding of spring malting barleys. But the main objective of this review is to pull together different aspects of what we know about winter-hardiness from the seemingly most essential aspect, which is survival in the field, to molecular genetics and gene regulation, and then finish with ideas that might help further our insight for predictability purposes.

## 1. Contextual Information for Understanding Malting Barley and Winter-Hardiness

### 1.1. The Life Cycle of Barley in Its Natural State

Writing a review on breeding winter-hardy malting barley might at first seem a straightforward topic to cover since barley exists in its natural state as a winter annual. However, spring, rather than winter barley has traditionally been used for malting and brewing. But there are now worldwide efforts being carried out by public and private breeders to develop winter barleys meeting modern malting quality standards in order to insure a stable supply of high-quality malting barley in the face of climate change and increased disease pressure, particularly as a consequence of maize cultivation practices.

Modern-day barleys are the result of selection pressures put upon wild barley *H. vulgare* ssp. *spontaneum* by humans starting about 10,500 years ago [1,2,3]. In its natural environment wild barley is adapted to a winter type growth habit [1,3,4,5,6]. Typically seed mature during the hot and dry conditions that prevail during late spring and early summer, and then lie dormant in the soil until autumn rains induce germination [1]. During winter and early spring, which is the rainy season, vigorous growth occurs, and this is followed by flowering and seed set [1]. Selection of the spring growth habit is thought to have been an important prerequisite to the radiation of barley into other regions of the world because it could then grow and mature in environments where winter temperatures were too cold for winter survival, or not cold enough to vernalize [3]. Consistent with this notion are findings that the alleles eliminating the requirement for cold temperatures to induce the transition from the vegetative growth phase to the reproductive growth phase, otherwise known as vernalization, are absent from wild barley [7]. Nonetheless, in regions distant to where wild barley naturally occurs, barley has still been cultivated as autumn-sown winter annuals, particularly in mid-latitudinal regions including North Africa, Europe, Nepal, China, and Japan [3,8], but through reproductive isolation and accentuated by the increased generation cycle frequency of modern plant breeding, spring barley actually now forms a subpopulation within *H. vulgare* ssp. *vulgare* [5,9,10].

*H*. *vulgare* ssp. *spontaneum* is indigenous to South West Asia. It occurs naturally in the area around the eastern Mediterranean to the eastern portion of the Iranian Plateau [3]. Within this region, one or more domestication events led to the first cultivated forms, *H. vulgare* ssp. *vulgare* [2,3,11,12,13,14,15,16,17,18,19]. One hypothesis is that there are two domestication centers, one in the Fertile Crescent and a second at the eastern edge of the Iranian Plateau [14,16]. From these epicenters barleys were carried by humans into other parts of the world, eventually reaching Northern Europe 6000 years ago and China 3000 years ago [3]. Population genetic evidence indicates that the European barleys are probably derived primarily from domestication events occurring in the Fertile Crescent, and that the Central and East Asian barleys are likely derived primarily from domestication events occurring at the eastern edge of the Iranian Plateau [14,16]. While these are believed to be the most likely paths, more recent genetic fingerprinting data indicate that the cultivated barley genome is actually a mosaic assembled from multiple wild barley, *H*. *vulgare* ssp. *spontaneum* populations indigenous to distinct geographic regions [20].

### 1.2. Malting Barley

When a farmer judges a wheat or barley variety for its worth, the biggest determining factor is likely to be yield. How many bushels per acre or tonnes per hectare will I get? When a brewer judges malting barley for its worth, a major determining factor is also likely to be yield. Except in this case yield refers to how much malt extract or barrels of beer will this pound of malt yield. These statements are an oversimplification but provide a starting point for what defines a malting barley because there really are no absolutes. The American Malting Barley Association provides guidelines for North American malting barley breeders (https://ambainc.org/amba-publications/guidelines-for-malting-barley-breeders/ (accessed on 08 July 2021)), noting that there is no one ideal variety because the end use may be different.

Several traits of key importance for acceptance as a malting barley include kernel weight, grain plumpness, grain protein, alpha amylase activity, diastatic power—the total enzymatic capacity to break down starch—and fermentability because these are associated with yield of malt extract in the brewhouse. Also of importance is β-glucan and the ability to hydrolyze β-glucan. β-Glucan is the primary structural component of barley endosperm cell walls, and its breakdown during malting enables amylases and proteases synthesized in the aleurone to gain access to their starch and protein substrates in the endosperm. Many traits of critical importance for malting quality and their molecular genetic underpinnings are addressed in detail in a review by Fox et al. [21]. These traits are also greatly affected by the environment in which a particular barley variety is grown. For example, drought or high temperature during grain fill drives up protein levels, which reduces extractable malt per gram malt. The environment can also alter the β-glucan composition in ways that drive up viscosity and drive down filterability [22,23,24,25]. These alterations are not well understood and could arise as a result of altered breakdown products of intermediate size. A malting quality barley therefore must perform such that it can be relied upon to give malt extract levels at some acceptable standard measure for the end user.

Malting and brewing qualities can be influenced by gene alleles that encode thermostable forms of enzymes [26], that allow greater clarity of the finished product [27], extend finished product shelf-life [28], and allow cleaner distillation [29]. Thermostable beta amylase gives the maltster and brewer greater flexibility when passing through the different temperate steps in the starch degradation processes. LOX-less eliminates lipoxygenase, an enzyme that creates precursors implicated in the staling of beer and the reduction of foam stability [28]. In a modern malting barley variety, these desirable qualities have been amalgamated together through selection and breeding over many generation cycles.

The challenge for breeders is that many of these traits are affected by independent loci dispersed across the genome and for many of the traits the marker trait associations are often not robust enough to use in a predictive way. In the breeding of commercial malting barley cultivars for the UK, molecular breeding tools have come into question as to whether they offer a means to increase extract or improve malting quality above that achievable through traditional phenotypic selection [30,31]. The environment in which the variety is grown can also have profound impact on the manifestation of malting quality traits. Recently Smith and colleagues [32] carried out a genome wide association study of malting quality across eight U.S. malting barley breeding programs. A key finding is that many of the regions of the genome that affect desirable trait qualities were unique to the breeding program. Because each breeding program typically works to develop varieties for its specific region, these data suggest a large component of the final manifestation of many malting quality characters are heavily influenced by the environment. At the genetic level, this appears as genetic drift in the breeding populations of individual programs, again likely due to selection in specific environments [33].

Once a variety is adopted by the end user there may also be a preference to stick with that variety because the malting and brewing conditions are fine-tuned to work with the nuances of that variety, whereas working with another variety may require adjustments to the malting and brewing procedures to obtain that ideal quality in the product. Often a variety will remain in cultivation for many years, sometime 10–20. This can be seen at the AMBA web site under know your malting barley varieties, which shows when each line was first recommended by AMBA (https://ambainc.org/wp-content/uploads/2021/04/KYMBV.pdf (accessed on 08 July 2021)). 

One obstacle faced by a barley breeder trying to develop new malting barley varieties—be it winter or spring—is the desire to maintain allelic combinations across the genome that are highly favorable for malting and fear of losing that malting quality when these allelic combinations are disturbed. Thirty years ago, in outlining a breeding strategy to develop improved varieties, Martin et al. [34] wrote “because of strict quality standards set by the industry, breeders have developed cultivars for malting by utilizing parents with good malting quality and introgressing other traits such as pest resistance in small increments over time, for fear that intercrossing with germplasm with poor malting quality would disturb already existing favorable gene combinations.” The scale of this idea is further exemplified by a statement Eslick and Hockett [35] made that they referred to as the “plant breeders dilemma.” Using the example of two barley varieties that differ in 10 traits, they show that over one million plants would need to be grown in the F_2_ generation to recover that single individual possessing the desired allelic combinations at 10 loci, assuming those 10 traits were each explainable by its own single locus. And that if those were planted one foot apart in a single row then one would need to walk nearly 200 miles to visually inspect each plant. And, if fate would have it, through some random act of nature, that one plant could very easily have been eliminated. The point is that there is reluctance to cross a good malting quality variety with a variety from a very different germplasm pool just to bring in another desired agronomic character. This lays out the daunting numbers faced when trying to combine the best qualities of a non-malting winter-hardy barley line with an elite spring malting barley.

### 1.3. Origin of Malting Barleys

Barley used for malting and brewing in Europe and the UK is traditionally carried out using two-row spring barley varieties, most of which trace to landraces cultivated in regions around the brewing centers of Bavaria, Moravia, Scandinavia, and the UK [36,37,38,39]. The Moravian landraces, collectively referred to as the Hanna (Haná) varieties, played a key role in contributing to the pedigrees of many breeding programs, as single plant selections from those landraces were also made in regions outside Moravia, and crosses to these selections or Hanna varieties were made by others [37,39]. ‘Binder’ and ‘Hannchen’—selections from Hanna landraces made in Denmark and Sweden respectively, are founding lines in many breeding programs. Even the two-row foundation germplasm in the U.S. originated from Moravian material. ‘Betzes’, one of the first two-row malting barley lines introduced into the U.S., results from ‘Bethges II’ × ‘Bethges III’, two selections from Moravian landraces. The same is true for ‘Moravian’, a line introduced into the United States by the Adolph Coors Brewing Co.

A detailed account of the progress in malting barley breeding carried out in the Czech Republic that begins with the earliest selections made from landraces in individual farmer’s fields can be found in Psota et al. [40]. A similar account documenting progress made in spring barley developed for cultivation in England and Wales is provided by Riggs et al. [41], while Russell et al. [42] provides a view into the narrowing of genetic diversity that occurred over time with this germplasm. Schittenhelm [43] assesses the morphological and agronomic changes that occurred in the barleys developed in Germany over an 80-year period. Across all of these programs the general trend is increased yield, and this yield increase is associated with changes in the individual character traits that include shorter plant height, increased straw strength, increased tillering, increased heads per plant, and increased weight of the grain kernel.

In the U.S., six-row barleys have also been used for malting and brewing, although they are now being phased out as the entire brewing industry moves to using exclusively two-row barleys (six-row vs. two-row refers to the structure of the spikelets on the rachis. Both have two-rows of main spikelets running down opposing sides of the rachis, each main spikelet having two laterals, but in two-rows the female laterals are sterile while in six-rows they are fertile, which gives rise to a threefold increase in seed numbers). These six-rows derive from landraces collected in North East Asia and have been referred to as the Manchuria-Ontario Agricultural College 21 (OAC 21) Oderbrucker group. They are thought to have first been introduced into the U.S. about 1861 and exhibited natural adaption to cultivation in the humid climate of the Upper Mississippi Valley and the Great Lakes regions [44]. They possessed a long lax spike which helped drying of the grain after rain, lessening grain disease [44,45,46]. They also possessed a sufficient level of dormancy so as to greatly reduce preharvest sprouting in the wetter climate. Because these lines also possessed high diastatic power, they were highly favorable for adjunct brewing in which starch sources other than malted barley are added during the brewing process.

A historical look at malting barley development in the U.S. up to 1973 can be found in Peterson and Foster [47]. Wych and Rasmusson [48] detail the gains made with each subsequent six-row variety release from the University of Minnesota and North Dakota breeding programs starting with the first Manchuria introductions. Essentially, the same advances made in breeding the six-rows parallel advances made in the two-rows. And perhaps counter to what might be predicted, significant gains in yield, lodging resistance, and malting quality were made despite the narrow genetic diversity of this germplasm (for fear that wide crosses would lead to loss of malting quality). Also of note is that the first winter barley released in the U.S. for malting, ‘Wintermalt’, is an offspring of ‘Traill’, an early line out of the North Dakota State University (NDSU) program, which was crossed to the winter-hardy feed barley ‘Hudson’ developed by Cornell [49].

The remainder of this review focuses on physiological and genetic analyses of winter-hardiness, the history of winter malting barley breeding, our current molecular genetic understanding of two of the key genetic components affecting winter-hardiness and finishes with some ideas on how we might further our understanding of winter-hardiness for predictivity purposes in breeding.

### 1.4. Winter-Hardiness and the Winter Growth Habit

Winter-hardiness embodies the capacity of plants to survive winter [50]. For cereals of the Triticeae, winter-hardiness implies the cultivation practice in which planting occurs in autumn and plants pass through winter prior to flowering and grain production the following spring. To complete this cycle these cereals must possess a level of low-temperature tolerance that enables them to survive the minimum temperatures in a given region, at different times during winter, and over extended periods [51]. Freezing tolerance is not a constitutive property however, rather it is induced or gained by exposure to low nonfreezing temperatures, a phenomenon known as cold acclimation [52]. Cold acclimation, freezing tolerance, and winter-hardiness are also strongly correlated with the reproductive state of the plant. Plants in a vegetative phase are better able to survive freezing temperatures than plants in a reproductive phase, and as the plant transitions from the vegetative to reproductive phase it gradually loses its capacity to cold acclimate and survive freezing [53].

The cereals of the Triticeae are generally classified as either having a winter growth habit, a spring growth habit, or an alternative growth habit. Winter growth habit cereal types are those that require vernalization to induce flowering, while those not requiring vernalization to flower are spring types. The alternative growth habit is somewhat of a gray area, and has been given different names, including alternative, intermediate, and facultative [54,55]. A current working definition is that facultative barley types have no vernalization requirement, mature when planted in the spring, and are as winter-hardy as winter types [54]. Some of the experiments leading to these classifications are elaborated on in more detail in the “Genetic analysis of winter-hardiness” section that follows later.

### 1.5. Regrowth in Spring Depends on Producing New Roots Following Extreme Cold

In many regions of the U.S. Midwest the ground freezes by mid- to late-November. Typically, there are only six to eight weeks of growth before all visible signs of above ground growth cease. Barley plants turn brown following a period of subzero temperatures, giving the appearance they are dead. In spring the plants will “green up” and continue to maturity if they have survived the winter. Survival however is highly dependent on soil temperature, soil moisture, the level of snow cover, and pathogens that may be present [56]. Of these, soil temperatures may be the most critical alongside the plant’s capacity to generate a new root system upon the return of favorable growth conditions in spring if roots were killed by soil temperatures that crossed a critical threshold.

Studies by Olien [57] indicate that roots near the barley crown and in the vascular transition region of the crown are more sensitive to freezing temperatures than are leaves and apical meristems. Warnes et al. [58] tested 25 winter barley varieties for their ability to regrow after freezing crown sections prepared from field grown plants allowed to grow for 8–10 weeks. In this experiment, and in a second in which 25 populations of field grown recombinants were assessed [59], 150 plants from each line or population were dug up; the soil, shoots, and roots were removed, leaving behind ~4 cm section encompassing the crown region, which was then frozen to −8.9 °C for a prescribed period. The crowns were then replanted and assessed for regrowth over the following three weeks. These experiments showed that the ability to produce a new root system was predictive of a line’s ability to regrow following the freezing treatment. Testing whether correlations existed between survival of the artificially frozen crowns with survival at multiple testing site locations across the U.S. indicated a strong correlation when plants were grown in a replicated plot format at the different locations [58]. In later experiments Chen et al. [60] showed that roots of winter wheat and winter rye are killed at temperatures below −8 °C, while their apical meristems are capable of surviving −20 °C to −30 °C, and the capacity for plant regrowth was highly dependent upon the capacity for initiating new roots.

### 1.6. Winter Barley and Winter-Hardiness in North America

Prior to 1920 the acreage of winter barley grown in the U.S. was of minor consequence [61]. Between 1934 and 1939 major increases in winter barley acreage took place in a region that stretched from the Atlantic coast to central Texas and Oklahoma, and included parts of southeastern Pennsylvania, the Piedmont Regions of Maryland and Virginia, the Ohio Valley, Missouri, and Kansas. The barleys grown in these regions are referred to as the Tennessee Winter types. Harlan [45] suggested they came from Switzerland or the Balkans, while Poehlman and Wiebe [61,62] considered the Caucasus region in addition to those suggested by Harlan. Recent genotyping data indicates they are closely related to winter accessions from Germany, Italy, and the Czech Republic [63].

The Tennessee Winter types are a diverse group because many selections based on local adaption and winter-hardiness were made, and these selections played a key role in stabilizing production across North America and to the continued increases in winter barley acreage [45,61]. By about 1940, states that had formerly grown primarily spring barley, including Illinois, Indiana, Kansas, Michigan, New York, Ohio, and Pennsylvania had transitioned to predominantly winter barley. By the late 1970′s more than 80% of the barley crop in these regions was winter barley [61].

During this period lines from around the world were evaluated for their winter-hardiness through field survival ratings taking place over multiple years in multiple locations [62,64]. Studies carried out in Missouri showed that the top-ranked lines were invariably the Tennessee Winter types, and these were followed by accessions from Korea, then accessions from Western Europe and the Caucasus [62,65]. Similar rankings were noted by Rohde and Pulham in Nebraska [66].

A much more expansive study, coordinated by USDA researchers Wiebe and Reid [64] that spanned the period 1937–1956, involved testing 204 genotypes at 111 sites across North America. Normalizing the survival of each line at each site location to three varieties included in all tests across all years, the authors ranked each line for survivability. The top 10 winter-hardy lines from this 20-year study are listed in Table 1. Four of these were selections from landraces or selections from farmers’ fields, three of which are classified as Tennessee Winter types while the fourth line, NE 62434, was a selection from a Korean landrace [61,64,65,67,68]. Six on this top ten list result from crossing, including the top two winter-hardy lines, ‘Kearney’ and ‘Dicktoo’, although neither has a known pedigree. A conclusion from this work was that it was possible to breed for increased hardiness by selecting under environments conducive to differential killing.

More recently, in a study carried out by Hayes and colleagues, winter survival of nearly 1000 accessions was assessed when grown over two seasons on three continents: at 13 locations in 2013–2014, and at 11 locations in 2014–2015 [63]. This study involved 21 barley breeding and genetics programs from around the world, each of which contributed germplasm, including cultivars, landraces, and advanced generation breeding lines from their respective programs. Each line was grown at each location as a single 1-m row and its winter survival was assessed against a set of three replicated controls in a Type II modified augmented design. Notably, the top two lines in the winter survivor category are MO B475 and Omugi Kauru Pori; the former the offspring from a Admire × Missouri Early Beardless cross (Tennessee Winter types), and the latter a Korean Landrace.

### 1.7. Breeding Winter Varieties of Cereals Traditionally Grown as Spring-Summer Annuals

Shortly after the “rediscovery” of Mendel’s work we learn of efforts to introduce the traits possessed by spring varieties of wheat and barley into their winter counterparts. In 1902 W.J. Spillman of the Washington Agricultural Experiment Station, one of four individuals credited with rediscovering Mendel’s work, describes the situation with wheat farmers in eastern Washington [69]. These farmers would plant the less hardy spring wheats in the autumn knowing that if they survived winter, then their yields would be much higher than if the same variety had been sown in the spring. The risk was that the autumn-sown spring variety crop did not always survive winter because they were less winter-hardy than their winter counterparts. Another advantage to an autumn sowing included earlier maturity, which distributed the farm workload more evenly. Yet, winter survival was the lurking problem. While there were winter-hardy types, they had a greater propensity for grain shattering, susceptibility to lodging and disease, and inferior milling and malting qualities. In comparison the agronomic traits such as strong straw and lodging resistance, and end-use traits of value, such as milling quality and malting quality, were superior in the spring growth habit forms.

### 1.8. Winter Malting Barley Development—Early Efforts

Early efforts to breed winter barley for malting trace to the geneticist and plant breeder E.V. Tschermak-Seysenegg at the University of Agricultural Sciences (Vienna, Austria) banother individual credited with rediscovering Mendel’s work. Around 1908 Tschermak-Seysenegg began making crosses with the two-row Hanna spring malting types to six-row winter barleys and to a two-row winter barley developed by a predecessor [70]. The offspring were then backcrossed to the winter two-row. The resulting barley was winter-hardy, lodging-resistant, had lower grain protein content, and received 2nd place (out of 370 entries) in an Austrian Brew Barley exhibition [70].

In 1932 G.D.H. Bell at the Plant Breeding Institute in Cambridge (UK) who had been breeding spring malting barley, made a directional change in his program to breed winter malting barley. In describing this directional change, Bell states the finest malting barley produced in the British Isles often occurred with an autumn sown crop using spring varieties—if they survived the winter [51]. Bell also gives many of the rationales given by Spillman for an autumn sown crop and the need for winter-hardy varieties. However, no true two-row winter barleys were cultivated on the British Isles, so he obtained Tschermak’s and Stadler’s A two-row winter lines from Bavaria. (Bell notes that Stadler’s A was a contaminant in a Bavarian winter wheat field, in contrast to Tschermak’s, which was a directed breeding effort). In evaluating the potential of these lines Bell describes the strengths and weaknesses of both, and states that neither met malting quality specs. A strength of Tschermak’s was its large thin husk, which is desirable for malting and brewing, whereas its weakness was its tendency towards lodging, while the strength of Stadler’s A was its bulky strong straw while its weakness was its thick husk. Thus, he began crossing the leading English two-row spring malting varieties to the two Bavarian winter barleys, and several years later to ‘Carstens’, another two-row winter barley also developed in Germany [51]. (Carstens = Eckendorfer Mammut (6r w)//Friedrichswerther Berg (6r w)/Svalofs Primus (2r s)). In 1943 he released ‘Pioneer’, a winter-hardy offspring from one of these crosses that resulted from Tschermak × Spratt-Archer, the latter, which was an offspring between ‘Spratt’ and ‘Archer’, two English landrace selections.

**Figure 1:**
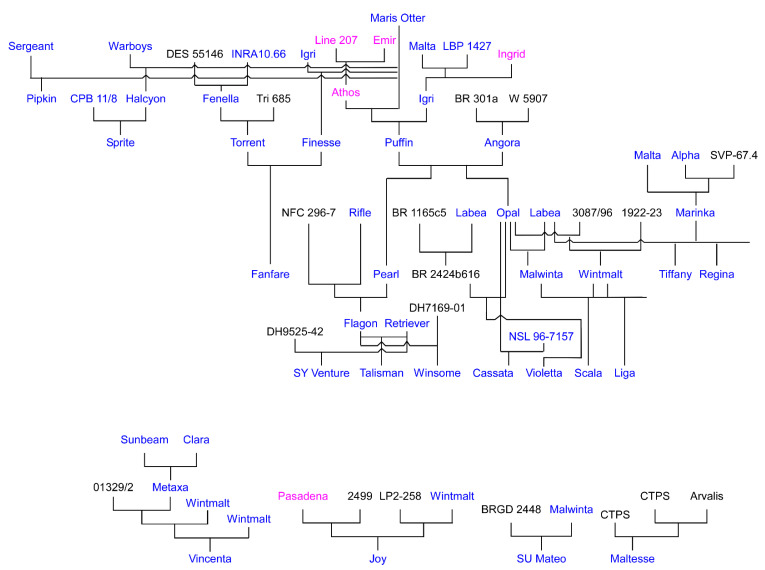
Pedigree tree of winter malting barley varieties grown in the UK and Europe, and those marketed in the U.S. Blue text are winter lines, pink text are spring lines, and black text are lines of uncertain growth habit that are breeder-specific.

The great success story was yet to come however from the cross between Pioneer and ‘Proctor’, which resulted in ‘Maris Otter’. Today, ‘Maris Otter’ is an iconic winter malting barley. Released in 1965 and recommended that same year for malting and brewing by the UK National Institute of Agricultural Botany (NIAB), it is still sought out by many brewers because of its superior malting and brewing qualities [31,71,72]. For example, Independent Craft Brewer Boston Beer Company uses ‘Maris Otter’ in their Extra Special Bitter, Samuel Adams Session Ale. Following the release of ‘Maris Otter’, the UK has had a continued development of winter malting lines. ‘Maris Otter’ is also the founding parent in the pedigree of nearly, if not all, winter malting barley cultivars now grown across Europe and the UK, and most recently, those now being marketed in the U.S. (Figure 1).

About 15 years after ‘Pioneer’ was released a great interest in winter malting barley arose in the U.S. because of an increased demand for malting barley by the brewing industry. Consequently, J.M. Poehlman, the barley breeder at the University of Missouri 1936–1980 entered a collaboration with Anheuser-Bush Inc. to evaluate the malting quality of Missouri germplasm. Up until this point Poehlman’s breeding program had focused on developing winter-hardy, disease-resistant, six-row feed barley, primarily with the Tennessee Winter germplasm [62,73,74]. At the same time (1961), the Malting Barley Improvement Association (MBIA), which later evolved to become the American Malting Barley Association (AMBA), provided three two-row winter barleys for testing, including ‘Carstens’, ‘Tschermak’, and ‘Pioneer’. That same year Poehlman also obtained an additional 68 two-row winter accessions from the USDA world collection. Over the course of the 1961–1962 and 1962–1963 field seasons these two-row lines were evaluated for their winter survival. Only ‘Tschermak’ and ‘Carstens’ showed any measurable level of winter survival and neither was as winter-hardy as Poehlman’s six-rows. ‘Pioneer’ was eliminated the first season because it matured too late [73].

**Figure 2:**
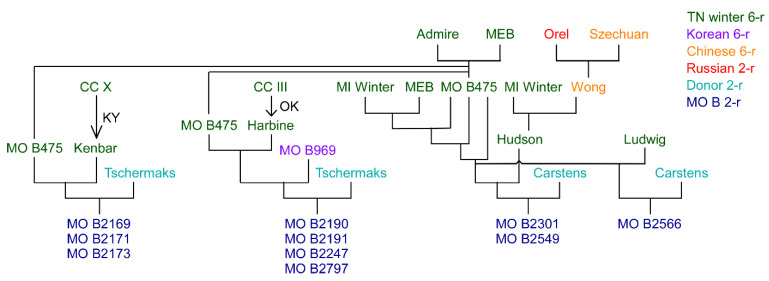
Pedigree of the two-row winter lines developed by Poehlman. ‘Kenbar’ and ‘Harbine’ are derived from Composite Cross X (CC X) and CC III, respectively. Composite cross populations were created by crossing a set of parental lines to one another in all possible combinations. Equal quantities of F_2_ seed from each F_1_ were then bulked and sent to barley breeders throughout the U.S., who then made selections in their environments. For example, CC III (CI 5530) was produced from crosses between 13 barley accessions, including winter, facultative, and spring types [64,75].

Poehlman crossed ‘Tschermak’ and ‘Carstens’ to his six-rows, leading to advanced selections that exhibited better malting-quality characteristics and which also possessed winter-hardiness levels approaching his six-rows (Figure 2) [74]. Poehlman retired in 1976 and these lines did not make it into commercial cultivation. Nonetheless he deposited these recombinants with the U.S. National Plant Germplasm System (NPGS) around the time of his retirement in 1976.

**Figure 3:**
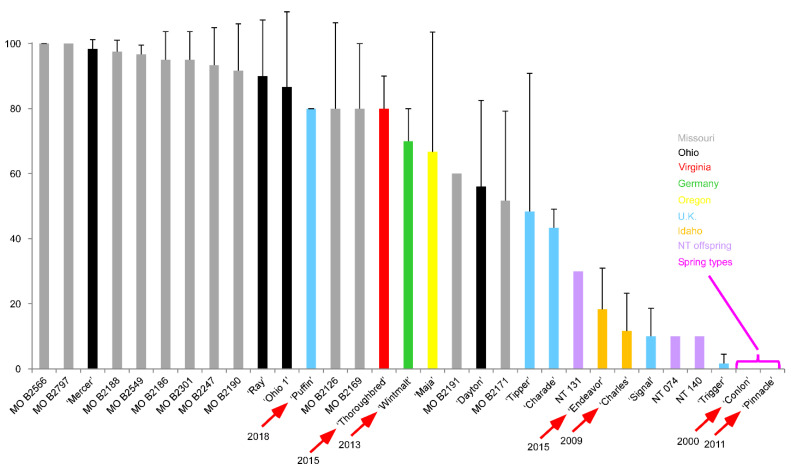
Percent survival 2013–2014. Plants were grown in 1.5 m × 3 m plots, planted seven rows across spaced 19 cm apart per plot in Wooster Ohio. Values are the percent living green plants relative to the total living + dead (brown) plants counted in May 2014 following green up per plot. Error bars show stand counts from three independent plots planted in randomized complete block design, except MO B2191, MO B2797, and the three N × T lines, which were each in a single plot. Color coding is used to represent material from different breeding programs. Red arrows identify AMBA-recommended malting barley lines and the year first recommended.

About 2008 this author took interest in the accessions that Poehlman had deposited with the U.S. NPGS. This interest was driven by findings that the *C-repeat Binding Factor* (*CBF*) genes at *FROST RESISTANCE-H2* (*FR-H2*), which is described in detail in sections that follow, were expressed to extremely high levels in ‘Admire’, the founding parent in Poehlman’s breeding program. Previous work also revealed that expression levels of the *CBF*s at *FR-H2* were in part dependent upon their allelic state [76]. Seeking to test whether this genetic association held for the ‘Admire’ allele and lacking a biparental population with ‘Admire’ as a parent, I began assembling all lines deposited by Poehlman with the U.S. NPGS and increasing this seed by single seed descent. Interest in Poehlman’s two-row material was also driven by knowledge that the craft brewing industry was seeking local sources of malting barley, and that Ohio had an ideal environment to produce high quality malting barley from winter genotypes, yet no program was working to evaluate or develop winter malting barley for the region. Thus, my lab also began putting Poehlman’s lines into the field and evaluating them alongside other two-row-winter lines that were either elite modern winter malting barleys or had at one time been a commercial winter malting barley variety. We also started making crosses between these two-row Missouri Barley (MO B) lines and these other malting lines. A subset of this material was in the field the 2013–2014 season when the extreme weather event, the 2014 North American cold wave, known as the “polar vortex” hit. Because the first wave of extremely cold air temperatures in January that dipped to about −25 °C on several consecutive nights occurred in the absence of snow cover, the winter proved to be one of those rare test winters which resulted in observed pronounced differences in survival across lines (Figure 3) that in a “normal winter,” would all have shown 100% survival.

While this is only one season of survival data following a test winter, the data show a skewing towards the MO B lines. The other lines in the top group are three lines previously selected for cultivation in Ohio: ‘Ray’, the product of a breeding effort in the late 1980s, while ‘Mercer’ and ‘Ohio 1’ are two six-row landraces collected from Ohio farms in the early 1940s and deposited with the NSGC.

**Figure 4:**
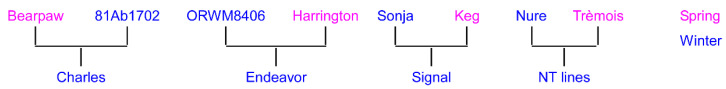
Crosses giving rise to lines exhibiting poor survival in Ohio the 2013–2014 test winter.

The winter lines exhibiting poor survival included ‘Charles’, ‘Endeavor’, ‘Signal’, and the three N × T lines (Figure 4). Each of these lines is derived directly from a winter × spring cross. The other low winter survivor ‘Trigger’ may also be from a winter × spring cross, but it was selected out of a composite F_2_, in which F_2_ seed from multiple different crosses of unknown parentage were bulked.

### 1.9. Genetic Analysis of Winter-Hardiness

In the first decade of the 20th Century researchers began making crosses between spring and winter types to understand the inheritances of growth habit and winter-hardiness. Phenotypic scoring to assign winter vs. spring categories to the offspring entailed field experiments at latitudes that ranged from 37.3° N at Suigen Korea by N. Takahashi, to 51.6° N at Saratov Russia by N.I. Vavilov [77,78]. The phenotypic screens of the resulting populations led to growth habit classifications of the recombinant offspring as (1) true winter, (2) true spring, (3) intermediate, and (4) pseudo-winter. Qualification for true winter meant the recombinant lines failed to mature when spring-sown and that they survived the winter when autumn-sown. Spring growth habit was qualified by maturation when spring-sown, and failure to survive the winter when autumn-sown. Intermediate types that matured when spring-sown and survived the winter when autumn-sown are now referred to as facultative types [54,55]. The pseudo-winter growth habit category was defined by recombinants that failed to mature when spring-sown and failed to survive winter when autumn-sown [77].

Early efforts to genetically characterize winter-hardiness in barley are summarized by Reid [79]. Several different inheritance schemes were proposed that ranged from complete dominance to complete recessiveness when winter × winter crosses were carried out. However, when winter × spring crosses were carried out, obtaining recombinants possessing the winter-hardiness level of the winter parent were the exception and frequently not recovered. Reid [79] also assessed winter survival in offspring derived from crosses between five winter barley lines and five spring barley lines. Included as winter parents were some of the top lines from the 20-year winter-hardiness study including ‘Kearney’ and ‘Dicktoo’. Most recombinant lines were eliminated by winterkill in the F_2_ and F_3_ generations; at the F_4_ a fraction of recombinant lines were recovered that exhibited survival levels approaching that of the winter-hardy parent when tested at multiple locations across the US. [79]. Using doubled haploid (DH) recombinants from one winter × four spring lines Doll [80] observed that the recombinants requiring vernalization comprised about 25% of the offspring and those lines exhibited 90% or better survival over the 1962–1963 winter in Denmark, while the lines not requiring vernalization exhibited about 60% survival.

Warnes and Johnson [59] also assessed survival in 25 different winter × winter crosses at Lincoln, Nebraska. Parental lines used in these crosses were categorized as hardy, moderately-hardy, and non-hardy, depending on how they survived the Nebraska test winter of 1965–1966. The offspring from these crosses were then rated for percent survival relative to that of the hardier parent used in each respective cross. In the offspring from hardy × hardy, most recombinant populations showed survival that paralleled that of the parents. In comparison, recombinants from the hardy × mod-hardy exhibited survival percentages ranging 35–75%, while recombinants from the hardy × non-hardy exhibited survival percentages ranging 10–28% that of the winter-hardy parent [59].

### 1.10. Molecular-Genetic Analysis of Winter-Hardiness

The first molecular marker genetic study of winter-hardiness in the Triticeae cereals was carried out in barley using a DH population developed between ‘Dicktoo’ and ‘Morex’ [81]. ‘Dicktoo’ is a winter-hardy line of unknown pedigree from a North Dakota breeding program [82] and ‘Morex’ is a spring malting barley from the University of Minnesota breeding program that provided *mor*e *ex*tract than that of any other midwestern six-row barley [83]. The Dicktoo × Morex (D × M) DH offspring were scored for measurable traits associated with winter-hardiness, including winter-survival at an Oregon location and a Montana location, regrowth following controlled freezing assays in growth chambers, and fructan levels in the crown of field grown plants; fructan being a fructose polymer that increases when cereal plants are cold acclimated and which shows a strong correlation with freezing tolerance levels of the crown [84]. Also measured was the time to flowering under a 24 h photoperiod. This analysis revealed a 21 cM interval on 5H that explained significant differences for these traits [81]. (In the original paper the chromosome was identified as chromosome 7, which was subsequently renumbered to 5H to be consistent with the syntenic relationship across other Triticeae [85].) Subsequent mapping efforts with more markers resolved the D × M winter-hardiness Quantitative Trait Locus (QTL) as being coincident with *VERNALIZATION-H1* (*VRN-H1*), one of two key loci determining the vernalization requirement [86].

About ten years later in an independent study, and using a Composite Interval Mapping strategy, Pecchioni and colleagues identified two QTLs on Chromosome 5 in a cross between ‘Nure’, an Italian winter feed barley and ‘Trèmois’, a French spring malting barley [87]. One of the key strategies used involved expression QTL analyses, in which levels of two proteins, which had previously been found to differ substantially between winter-hardy and non-winter-hardy barleys, were measured in the segregating population.

These two loci have now been repeatedly identified in barley and are referred to as *FROST RESISTANCE-H1* (*FR-H1*) and *FR-H2* [88,89,90]. Both reside on the long arm of chromosome 5 and at genetically co-linear positions in the genomes of barley, diploid wheat, and the three component genomes of hexaploid wheat [81,86,87,91,92,93,94,95,96,97,98,99,100]. These loci are referred to hereafter below as just *FR-1* and *FR-2* when a specific Triticeae species is not being discussed. The current thinking is that *FR-H1* is due to the *VRN-H1* gene and *FR-H2* is due to *CBF* genes. But these loci are complex, particularly *FR-H2* and we still do not have a complete mechanistic picture of how these loci are driving winter-hardiness. A third locus, *FR-H3* on chromosome 1H, has also been identified in a subset of populations [88]. Key steps that led to the identification of *FR-H1* and *FR-H2* is described in sections that follow.

### 1.11. Isolation of Genes Robustly Induced by Low Temperature in Plants

In the late 1980s the molecular isolation of genes whose transcripts were increased by exposure to cold temperatures, or dehydration stress, or salt stress were carried out in many plant species, some of which included barley, wheat, and Arabidopsis [101,102,103,104,105]. These studies were driven in part by the hypothesis that the encoded proteins played an important role in protecting the plant from the cellular dehydration imposed by these stresses. The genes and encoded proteins were given different names, based largely on the system the researchers were using and their means of gene induction, and these names include *BLT* (*barley low temperature*), *COR* (*cold-regulated*), *DHN* (*dehydration induced*), *LEA* (*late embryogenesis abundant*), and *WCS* (*wheat cold specific*). In many instances the same genes were identified via a different differential screen. Today we have some ideas and mechanistic insight as to how these proteins function to enhance resistance to cellular dehydration, principally by associating with the cellular endomembrane system, but there are still many unknowns [106,107,108,109].

### 1.12. Mapping Loci Affecting COR Expression and Freezing Tolerance in Wheat and Barley

In barley it had been recognized that RNAs for the cold-induced *BLT* genes accumulated to higher levels in winter genotypes (including the malting barley cultivar ‘Maris Otter’) than in spring genotypes [102]. In winter wheats the product of one such gene, the WCS120 protein was produced in levels that were so much higher than in the spring wheats the idea arose that breeders could use that phenotypic difference as a marker to score freezing tolerance in a segregating population [105]. Other experiments indicated that *COR14* and *COR75* transcripts appear at warmer temperatures and accumulate faster and disappear slower in winter cultivars relative to their spring counterparts [110,111]. Yet there was no evidence to indicate that the genes from which the *COR* transcripts emanated were substantially different between spring and winter types, or that there were gene copy number differences between winter and spring genotypes [102,104].

Pecchioni and colleagues exploited this phenotypic difference and used it as an expression QTL (eQTL), the strategy of which consisted of measuring the protein levels of two *COR* genes in each of the ~130 DH offspring from the Nure × Trèmois cross (N × T population) [87]. Finding two regions of chromosome 5 that explained significant differences in COR protein accumulation, they mapped a single barley EST (Expressed Sequence Transcript) whose BLAST annotation indicated it was a strong hit to the Arabidopsis *CBF* gene, right on top of the proximal QTL. However, neither region harbored the coding sequence for the two *COR* gene used in the eQTL strategy [87].

### 1.13. Identification of the CBFs in Arabidopsis

A key feature uniting many of the *BLT*, *COR*, *DHN*, *LEA*, and *WCS* genes is that they exhibit a robust and coordinated induction by cold temperatures [112]. Fusing the promoter of one of those *COR* genes to the β-glucuronidase reporter gene and transforming that back into *Arabidopsis* revealed that the *COR* gene promoter was cold temperature responsive [113]. Deletion analyses of two *Arabidopsis COR* gene promoters (*COR15a* and *COR78*) performed by independent groups, alongside nucleotide substitution through candidate motifs revealed the sequence motif TGGCCGAC in the *COR15a* promoter, identifying it as the C-Repeat, CRT, and the TACCGACAT motif in the *rd29A* (*COR78*) promoter, identifying it as the dehydration responsive element, DRE [114,115]. Both the CRT and DRE share the core nucleotide sequence CCGAC, which has come to be known as the CRT/DRE. This motif is conserved across plant taxa, occurring in many of the upstream regions of low temperature induced genes (including the *COR14b* gene used in the eQTL strategy used by Pecchioni and colleagues) suggesting that it is a universal cis-acting regulatory motif in plants for genetic programming in response to sensing low temperature and osmotic stress [116].

The CRT/DRE motif then served as bait in yeast one hybrid screens in which the principle was to identify proteins capable of binding to the CRT/DRE from screens of libraries of Arabidopsis proteins. These screens led to the isolation of *C*-repeat/DRE *B*inding *Factor 1* (*CBF1*) [117] and the DRE binding proteins (DREB) [118]. *CBF1* turned out to be one of three genes linked in tandem, in a *CBF1*-*CBF3*-*CBF2* head-to-tail cluster on chromosome 4 [119]. All three genes are induced within minutes of the plant’s exposure to low temperature, and their induction precedes *COR* gene activation [119]. When *CBF1*, *CBF2* or *CBF3* are overexpressed as a transgene, a constitutive cold acclimation phenotype is conferred in which accumulation of *COR* gene transcripts and freezing tolerance are greatly enhanced at normal growth temperatures [118,120,121]. Transcript profiling of the genome showed that nearly a third of the cold temperature responsive transcriptome is under regulatory control of the CBFs, and this is now referred to as the CBF regulon [122,123]. 80% of the genes comprising the CBF regulon harbor at least one copy of a CRT/DRE consensus sequence A/GCCGAC within 1 kb upstream of the coding sequence, making them likely direct CBF targets. [123,124].

CBFs are APETALA2 (AP2) domain containing transcription factor proteins, of which there are about 150 in the Arabidopsis genome. The CBFs are distinguished from other AP2s by the CBF signature sequences, short amino acid motifs that flank the AP2 domain and are required for binding to the CRT/DRE [125,126]. In Arabidopsis six AP2s meet this criterion. In contrast to the cluster structure of *CBF1*-*CBF3*-*CBF2* on chromosome 4, the three other CBFs reside as single genes. *CBF4* (*DREB1d*) resides on chromosome 5 and two additional CBF signature sequence AP2s, *DDF1* (*DREB1f*) and *DDF2* (DREB1e), are on chromosome 1 and separated by about 25 cM [124,127,128]. In one set of experiments *CBF4* showed rapid induction in response to drought and abscisic acid (ABA), but not by cold. In a different set of experiments, *CBF4* responded to salt stress but not cold, drought, or ABA [124,129]. Overexpression of *CBF4* also activates *COR* genes, and increases freezing and drought tolerance [129]. *DDF1* was identified through an activation tagging mutant screen for gibberellin-deficient mutants and exhibits a *dwarf and delayed-flowering* (*DDF1*) phenotype [127]. Its upregulation in the activation-tagged *DDF1* mutants results in higher levels of *RD29a* (*COR78*), and these *DDF1* overexpressors have significantly greater salinity tolerance than wild type plants [127]. Thus, these six *CBF* genes are rapidly but differentially induced in response to environmental conditions that impose a cellular dehydration stress upon the plant, and they activate overlapping if not identical sets of downstream genes that confer tolerance to cellular dehydration.

### 1.14. CBFs in the Cereals and Copy Number Variation

Given that CBFs affected freezing tolerance of *Arabidopsis*, an important question became what role did the CBFs play in crop plants. In early 2001 this author teamed up with the labs of Pat Hayes and Tony Chen at Oregon State University and later with that of Nicola Pecchioni at the CRA-Istituto Sperimentale per la Cerealicoltura in Fiorenzuola d’Arda, Italy to address this question and to gain a greater understanding of the nature of *FR-H2* and whether the *CBF* genes might be the underlying molecular basis of *FR-H2*. An initial objective was to isolate every *CBF* gene from barley, and essentially a no holds barred strategy ensued. Nine unique CBF sequences were identified from the NCBI barley EST database [130]. Using those as probes in screens of cDNA libraries constructed from cold-acclimated ‘Dicktoo’, and genomic libraries constructed from ‘Dicktoo’, ‘Nure’, ‘Morex’, and ‘Trèmois’ revealed even more *CBF*s. Twenty unique *CBF* genes from a single genotype were identified, which resolved into three distinct clades [86,99,100,130]. Genes in the HvCBF1 clade reside across the genome, while those in the HvCBF3/CBFIII and HvCBF4/CBFIV clades localize to *FR-2*, which is on the long arm of chromosome 5. A single genotype possesses at least 13 different coding sequences at *FR-2*, while some genotypes possess even more through increased copies of paralogs of those same 13 [100,130,131,132,133,134]. A framework physical map of the *FR-2* region was provided through sequencing large insert BAC clones from diploid wheat line DV92 [131]. Additional and higher resolution mapping in diploid wheat and barley alongside production of a physical map of the *FR-H2* region from barley cultivar ‘Morex’ positioned additional *CBF*s and showed a high level of gene order conservation between diploid wheat and barley [100,131,133,134].

Sequencing bacteriophage lambda genomic clones encompassing collinear regions of the genome from the four barley genotypes showed that there was even greater complexity: lines carrying the winter *vrn-H1* allele harbored more *CBF* coding sequences, and in greater incarnations than the lines carrying a spring *Vrn-H1* allele [100]. In the genomes of ‘Dicktoo’ and ‘Nure’, *CBF13* is a bona fide coding sequence but *CBF13* of the ‘Morex’ and ‘Trèmois’ genomes carries multiple inactivating disruptions, some of which are conserved between ‘Morex’ and ‘Trèmois’, and some of which are unique to each. Those data suggest a common pseudogenization event followed by accumulation of additional mutations along separate paths. Other differences that stand out include absence of *CBF10B* from ‘Morex’ and presence of the *CBF10A* and *CBF10B* paralogs as a tandem pair in ‘Dicktoo’, ‘Nure’, and ‘Trèmois’ [86].

**Figure 5:**
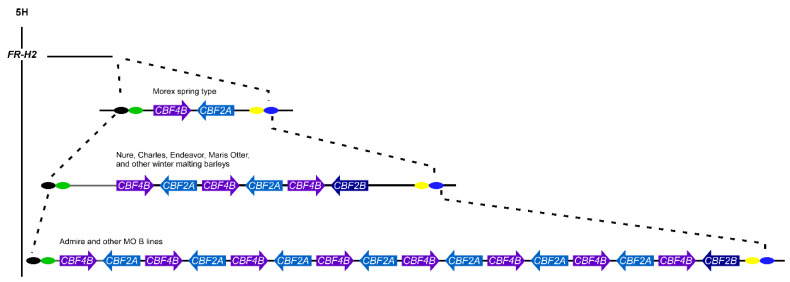
Illustration of the 22 kb *CBF2A–CBF4B* genomic region copy number variable unit. Top depicts the Morex-like spring allele. Middle shows the Nure allele as determined from sequencing BAC clones [137], which is predicted to be representative of current winter malting barley lines based on sequencing, DNA blot hybridization, pedigree, or any combination of these [100]. Lower allele depicts hypothesized Admire allele, which were estimated by DNA blot hybridization and following the structural organization pattern revealed by Mareri et al. with the centromere toward the north on the vertical illustration and left on the horizontal illustration [135,137]. Other MO B lines possess copy numbers that are intermediate between Admire and the Dicktoo-Nure allele [135]. The colored ovals are used to illustrate a common region across genotypes outside the iterated 22 kb *CBF2A–CBF4B* genomic region. For example, the green oval identifies a region containing a *Xba* I site that is common across genotypes having the Dicktoo-Nure allele, which is 6.0 kb from an *Xba I* site in the iterated region. Using a *CBF4* coding sequence probe on an *Xba I* digest in DNA blot hybridizations results in a single copy 6.0 kb MW fragment, and a multi-copy 9.3 kb fragment.

More surprising at the time was finding that the genomic segments encompassing *CBF2* and *CBF4* are tandemly duplicated in ‘Dicktoo’ and ‘Nure’, and the *CBF2A* and *CBF4B* coding sequences in adjacent genomic unit segments, which are about 22 kb in length, are identical to each other [100]. One hundred percent identity extends about 500 bp into the 5’ and 3’ flanking sequences. Only in the intergenic region do the unit segments become distinguishable, and even then, the intergenic regions are 97–98% identical [100]. DNA blot hybridization revealed that many cultivated winter barleys possess a Dicktoo-Nure type allele including ‘Maris Otter’ and other winter malting barley varieties [100]. In comparison ‘Admire’ showed 7–8 copies, while other MO B lines showed copy numbers that could be categorized as 1–3, 4–5, or 7–8 [135]. Francia et al. [136] estimate that there are at least ten copies of *CBF4B* and eight copies of *CBF2* in some lines. In comparison, the *CBF2* and *CBF4* genes of the spring genotypes ‘Morex’ and ‘Trèmois’ are present in single copies [100]. The gene structure of the spring genotypes is colinear with the *CBF2A*–*CBF4B* genomic region of ‘Nure and ‘Dicktoo’, but it is present in only single copy in ‘Morex’ (Figure 5; [100,134]). Like ‘Morex’, the spring barley ‘Trèmois’ also harbors only single copies of *CBF2* and *CBF4* gene paralogs but the genomic regions encompassing the ‘Trèmois’ genes are distinctly different from those of ‘Nure, ‘Dicktoo’, and ‘Morex’, exhibiting only partial collinearity [100]. Nonetheless *CBF2* and *CBF4* in these spring genotypes are bona fide coding sequences (CDS), i.e., they are not null alleles. Surveying a panel of diverse winter and spring barley accessions using DNA blot hybridization indicates the widespread phenomenon of *CBF2* and *CBF4* copy number differences discriminating winter and spring barleys [100,136]. *CBF12* and *CBF15*, and perhaps other genes are also present in duplicate form as identical or nearly identical paralogs in individual genomes, although there has yet to be evidence supporting haplotype associations with spring and winter alleles at *VRN-H1* [100,134].

The structural organization of *FR-H2* was recently refined through producing a BAC library of ‘Nure’ and sequencing clones harboring *CBF*s [137]. This is an important step, as a large insert library constructed from a winter genotype had not previously been available. This work provides a clearer picture of the copy number variable unit harboring the *CBF2A* and *CBF4B* genes and establishes that the single copy *CBF2B* gene resides at one terminus of the copy number variable unit [100,135].

While the basic structural unit underlying the Dicktoo-Nure (DN) allele seems to occur in many winter barley varieties, DNA blot hybridization indicate that there are at least three additional distinct “winter alleles” of *FR-H2* which have not yet been characterized at the structural level, including the ‘Kompolti korai’, Mumie Pori (MP), and MO B1252 alleles [100,135]. The Kompolti korai allele can be traced to landraces of Hungary, France, and Belgium [100]. Mumie Pori is a Korean landrace, as is Chae-Rae-Chang (PI 157659), both of which have the same allelic form. This *FR-H2* allelic form exhibits two *CBF2* paralogs, which appear to each be single copy, but it also appears to harbor increased copy numbers of *CBF14* in comparison to that of ‘Dicktoo’, ‘Nure’, and ‘Admire’ suggesting *CBF14* is present in multiple copies. The MP allele also possesses a *CBF4* paralog coding sequence, but the DNAs of the MP allele do not cross-hybridize with probes immediately 5′ and 3′ to the ‘Dicktoo’ and ‘Nure’ *CBF4* coding sequence, indicating their *CBF4* exists in a distinctly-different genomic environment [86,100,135]. The MP allele is also the allelic form that occurs in 88Ab536-B, a six-row winter barley line possessing excellent malting quality characteristics [100,138,139]. Many of the other *CBF*s at *FR-H2* also exhibit distinct differences with the DN alleles, but share commonalities with spring alleles, suggesting the DN and MP alleles may have each independently contributed towards extant spring lines [86,100,135]. This may help explain the association of allelic variants of *CBF14* with increased cold tolerance [140]. Fricano et al. [140] also note that there have been substantial reductions in diversity at the nucleotide and haplotype levels in cultivated lines over landraces, finding *CBF9* to be nearly monomorphic in the cultivated germplasm.

### 1.15. Winter-Hardiness and Its Connection to the Reproductive State of the Plant

In the 20-year winter-hardiness study carried out by Wiebe and Reid [64] ‘Dicktoo ranked #2. Yet when grown in a greenhouse without vernalization under a 24 h photoperiod it flowers just two weeks later than ‘Morex’, despite exhibiting the winter growth habit in the field [81]. As a result of this phenotype, ‘Dicktoo’ was eventually reclassified as facultative [54]. These phenotypic responses have been crucial in leading to a better understanding of the connection between winter-hardiness and the reproductive state.

At germination, genotypes or lines that are in the vegetative phase are typically of the winter growth habit, while genotypes in the reproductive growth phase at germination are typically of the spring growth habit [141,142]. In the case of winter genotypes, a period of exposure to low nonfreezing temperatures is required to cause the transition to the reproductive state, while this requirement is dispensed in spring genotypes. This difference between the winter and spring growth habit is controlled by three distinct genetic loci, *VERNALIZATION-H1* (*VRN-H1*), *VRN-H2*, and *FT1* (*VRN-H3*). The spring growth habit is manifest through a dominant spring allele at *VRN-H1* (*Vrn-H1*), which constitutively expresses *VRN-H1* resulting in a constitutive progression down the reproductive growth pathway. In comparison, the winter (and facultative) growth habit is manifest through the recessive winter allele at *VRN-H1* (*vrn-H1*), which does not accumulate *VRN-H1* transcripts until other endogenous signals or external cues including prolonged exposure to cold, long day photoperiod, or both trigger induction of its accumulation. *FT1* (*VRN-H3*) plays a role in promoting the reproductive growth pathway but can either require expression of *VRN-H1* first, or override this requirement depending on its allelic state [143]. The genetics and mechanisms by which the genes at these loci function are reviewed by Trevaskis and colleagues [144,145].

‘Dicktoo’ carries a *vrn-H1* winter allele at *VRN-H1* and a *vrn-H2* spring allele at *VRN-H2* [54,146]. The spring *vrn-H2* allele removes a repressor of *VRN-H1* expression under LD growth conditions [141,142]. The *vrn-H1* winter allele at *VRN-H1* restricts expression of *VRN-H1* in ‘Dicktoo’ until the critical minimum photoperiod of 12 h is reached [147]. Once *VRN-H1* transcripts begin to accumulate the plant begins a committed step towards the reproductive state, which also results in a progressive and substantial increases in *VRN-H1* transcript levels. This separation of the two growth phases is paralleled by levels of freezing tolerance and levels of *CBF* and *COR* gene transcripts: these are higher in plants maintained in the vegetative phase under short day (SD) photoperiod and are lower in plants grown under long day (LD) and in the reproductive phase [76,148,149]. Partitioning into the distinct growth phases by photoperiod is most-likely gated solely by the induction of *VRN-H1* transcript accumulation itself. Plants maintained under SD do not accumulate *VRN-H1* while plants growing under LD do [76,150].

Typically, winter barley (and winter wheat) is planted around the vernal equinox or after, when day length is decreasing. This date will vary across locations, depending on latitude and other seasonal temperature norms. Thus, the strong photoperiod gating of *VRN-H1* in ‘Dicktoo’ could conceivably limit its expression across a wide range of environments until the vernal equinox the following spring, ensuring the plant is maintained in the vegetative phase until that time. This mechanism would explain the superior winter-hardiness exhibited by ‘Dicktoo’.

However, this unique property of ‘Dicktoo’ is not necessarily indicative that all barley genotypes possessing the alternative growth habit are capable of surviving winter. Cockram [55] provides the different gene allele combinations capable of conferring the alternative seasonal growth habit, showing that the mutant *ppd-H2* allele at the *PHOTOPERIOD-H2* locus, which delays flowering under SD, will confer the alternative growth habit when in haplotype association with a spring allele at either *VRN-H1* or *VRN-H2*, if the other locus carries the winter allele [55]. In these instances, it is likely that that the allelic state at *FR-H2* and regulatory control over the *CBF* genes at *FR-H2* are key determinants of winter-hardiness.

### 1.16. Relationship between Expression of VRN-H1 and CBFs

Experiments in wheat and barley showed that a progressive reduction in freezing tolerance occurs as the plant makes the reproductive transition and that this parallels a decrease in the levels of the *COR* genes [149,151]. Later experiments revealed the *CBF* genes were deactivated following the reproductive transition and that mutants deleted for *VRN-1* continue to express the cold acclimation pathway genes under cold acclimating conditions [76,152]. Direct support for VRN-1 acting to repress *FR-2* comes from the work of Trevaskis and colleagues [153] who carried out chromatin immunoprecipitation-sequencing (ChIP-seq) with an α-VRN-1 antibody, revealing that the VRN-1 protein directly binds to the upstream regions of *FR-H2* genes *CBF2*, *CBF4*, and *CBF9*. In essence, *FR-1* is potentially epistatic to *FR-2* through a mechanism in which the VRN-1 protein binds the genomic regions around the *CBF* genes, leading to the deactivation of the *CBF*s and the pathways they control. Nonetheless exact details how this occurs are not known and there may be other mechanistic events preceding VRN-H1 binding to the *CBF* genomic regions.

**Figure 6:**
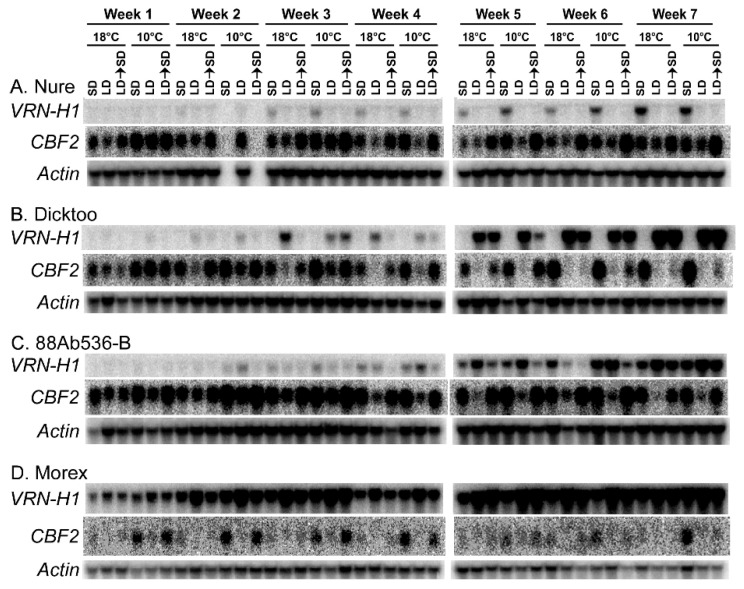
*VRN-H1* and *CBF2* expression in ‘Nure’, ‘Dicktoo’, 88Ab536-B, and ‘Morex’ under SD and LD photoperiod over seven weeks. Each lane was loaded with 7 μg total RNA isolated from crowns. Filters were hybridized together in the same solution to each respective probe. Plants were grown under a light/dark cycle of 8 h/16 h (SD) or 16 h/8 h (LD) using 650 μM m^−2^s^−1^ light. Tissue was harvested 6 h after subjective daybreak from the SD plants, and 14 h after subjective daybreak from the LD plants. At daybreak the day after the harvest of the 18 °C sample set, the growth chamber temperature was decreased to 10 °C. Tissue was then harvested that same day. The day following the temperature decrease to 10 °C, the growth chambers were returned to 18 °C. Concurrently a set of two pots of each genotype were transferred from the LD chamber to the SD growth chamber. This cycle was repeated for seven weeks. The lanes marked LD → SD are from a set of plants (two pots each genotype) that were moved from the LD chamber to the SD growth chamber five days prior to the 18 °C temperature tissue harvest. Transfers were made at weekly intervals starting the second week after germination.

To assess the expression pattern of *CBF2* over time relative to that of *VRN-H1*, the two genes were tracked over a seven-week time course in four genotypes: ‘Nure’ (winter and haplotype *vrn-H1*/*Vrn-H2*), Dicktoo and 88AB536-B (facultative and haplotype *vrn-H1*/*vrn-H2*), and Morex (spring and haplotype *Vrn-H1*/*vrn-H2*) (Figure 6). This experiment was carried out under SD and LD photoperiod. The single harvest point each week was timed to capture *CBF2* at peak expression during the circadian cycle, which occurs about 6 h into the subjective day (lights on) in plants grown under SD, and about 10 h into the subjective day in plants grown under LD [135]. Tissue was harvested from plants grown at normal growth temperatures (18 °C) and from plants subjected to a temperature decrease to 10 °C to test whether changes in *CBF2* levels occurred in response to the temperature decrease. From this experiment it is clear that *CBF* transcripts accumulate to much higher levels in the three genotypes possessing a winter *vrn-H1* allele (Figure 6A–C) than in the line having the spring allele (Figure 6D) at both temperatures. Other patterns of expression were more genotype specific. In ‘Dicktoo’ (Figure 6B) the contrasting expression pattern of *VRN-H1* and that of *CBF2* is readily observed. *CBF2* accumulates under SD and LD at week 1 but by week 2 begins to show decreased levels under LD. This is inversely paralleled by *VRN-H1*, which does not accumulate under SD, but steadily increases under LD. When *VRN-H1* transcripts accumulate to relatively high levels, *CBF2* transcripts do not or are greatly diminished. In ‘Nure’ (Figure 6A), *VRN-H1* is not initially expressed under SD or LD, but transcripts steadily increase each week under SD, which is consistent with *VRN-H2* acting to repress expression of *VRN-H1* under LD [144,154,155]. Within this 7-week time frame however there is no detectable reduction in *CBF2* transcript levels in ‘Nure’.

88Ab536-B is another facultative genotype that lacks a functional copy of *VRN-H2* [54]. In this genotype (Figure 6C), *VRN-H1* showed a progressive increase under both SD and LD photoperiods. The accumulation of *VRN-H1* under LD is slightly enhanced over that of SD. *CBF2* levels showed a correspondingly diminished levels under these same photoperiod conditions.

To test whether the effects of LD growth conditions established permanency in the transcriptome state, one set of plants of each of the four genotypes was transferred from LD to SD each week and allowed to grow for five days prior to being assessed for gene transcripts. In all four genotypes *CBF2* transcripts were expressed in the LD → SD set at levels comparable to the SD set through about the fifth week. At the 6th week, *CBF2* transcript levels began to show a noticeable decline for the LD → SD sets relative to the SD set in ‘Dicktoo’ and ‘Morex’. For 88Ab536-B this appeared to be delayed by about one week and for ‘Nure’ there was no noticeable decrease in *CBF2* levels in the LD → SD set through the 7th week. *VRN-H1* transcripts also showed a much higher level in ‘Dicktoo’ and ‘Morex’ earlier in the time course than in 88Ab536-B, while in ‘Nure’ *VRN-H1* transcript levels were comparatively much lower. These data indicate there is an inverse expression pattern between *VRN-H1* and *CBF2*, but there was no definitive absolute on vs. off demarcation pattern, suggesting there is overlap between the two genes within the crown tissue sampled.

The two facultative genotypes 88Ab536-B and ‘Dicktoo’ differed primarily in the *CBF2* transcript levels detected in the warm-grown plants. In ‘Dicktoo’ *CBF2* exhibited a trend of increasing levels at both temperatures over the time course under SD, particularly at the later weeks. In 88Ab536-B *CBF2* exhibited a more robust accumulation earlier in development, particularly at 18 °C. This increase in expression appeared to taper off by Week 7. In looking at this data it must also be kept in mind that 88Ab536-B possesses the Mumie Pori allele, and thus presumably only a single copy of *CBF2*, in comparison to ‘Dicktoo’, which possess three copies of *CBF2* as a consequence of the Dicktoo-Nure allele. These data suggest that under SD a greater activated state of the CBF programming system develops over time in ‘Dicktoo’, whereas it is in a more activated state earlier in development in 88Ab536-B.

In wheat *VRN-1* exhibits copy number variation [156,157], which is likely to affect expression levels once induced. Similar variation may occur in barley for *VRN-H1* as copy number variation is detected at other loci affecting the reproductive transition including *FT* in barley and *Photoperiod-B1* in wheat [143,156]. In an assessment of the growth habit of cultivated barleys from around the world, Saisho et al. [158] found there was a greater propensity for a vernalization requirement and a longer vernalization requirement in accessions from Northeast Asia in comparison to accessions from Europe. Notably, the greater entrenchment of the winter growth habit in the North East Asian accessions is attributed to loci other than the three known major genes affecting the vernalization requirement and flowering time, *VRN-H1*, *VRN-H2*, and *VRN-H3* [158]. Given our current understanding, it seems likely the winter-hardiness in Northeast Asian accessions detected by North American barley breeders half a century ago is due to allelic components maintaining plants in a vegetative phase during a critical window. These components potentially have been inherited by 88Ab536-B along with the Mumie Pori *FR-H2* allele.

### 1.17. CBFs Activate Other CBFs

Upon finding that the allelic states at both *FR-H1* (*VRN-H1*) and *FR-H2* affected *CBF* levels and that the genes of the winter barley ‘Nure’ were deactivated following vernalization, the first questions raised were whether this phenomenon also occurred for other barley genotypes and whether it was more widespread across the Triticeae [76]. In addressing these questions, it was revealed that many of the *FR-H2 CBF*s accumulated to much higher levels in ‘Admire’ than in other winter barleys [76]. Subsequent characterization of this phenomenon indicated that the *CBF2A*–*CBF4B* genomic region in ‘Admire’ exists in even higher copy numbers [135]. However, there is no evidence that any of the *FR-H2 CBF*s of ‘Admire’ differ in copy number from other winter barley varieties. Rather it appears that higher copy numbers of the *CBF2A*–*CBF4B* genomic region result in increased expression levels of *CBF2* and *CBF4*, which in turn induces increased expression of several of the other *FR-H2 CBF*s. Barley plants overexpressing *CBF2* show higher transcript levels of *CBF12*, *CBF15*, and *CBF16* [159] and ChIP experiments indicate *CBF2* is binding to the *CBF12* and *CBF16* promoters at normal growth temperatures [135]. Each of these genes harbor one or more CRT/DRE motifs in their upstream regions. Assessing a subset of the MO B lines related to ‘Admire’ by descent also indicated that *CBF12* and *CBF16* accumulate to significantly higher levels in lines having 6–8 copies than lines having 1–3 copies. This occurred at normal growth temperatures and was highly dependent upon the circadian clock [135]. More recently Mareri et al. [137] show that the single copy gene *CBF14* is also expressed to higher levels in lines possessing higher copy numbers of the *CBF2A*–*CBF4B* genomic region.

Thus, it seems one function of the *CBF2A*–*CBF4B* genomic region copy number variation is to simply effect transcript levels of the *CBF*s and their targets. Nonetheless this still leaves open the question of whether each of the genes at *FR-H2* has a more specific biological role and is critical for environmental adaptation. For example, lines having mutations affecting the DNA binding of *CBF12* indicate that it is critical [133], while genetic association and overexpression studies indicate that *CBF14* is critical [140,160]. Some may be dispensable, while others are not. *CBF13* is a bona fide coding sequence in winter barleys but it is a pseudogene in spring barleys [100]. And while *CBF2* and *CBF4* are present in multiple copies in the winter barley, they are still present as a single copy gene in the spring genotypes, which suggests that they may not be dispensable.

**Figure 7:**
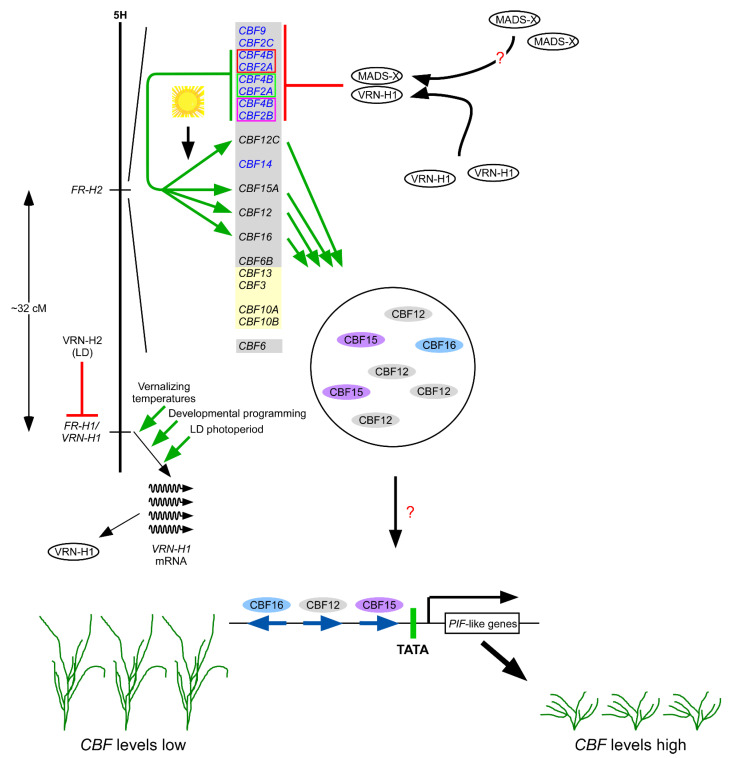
The genes at the *FR-H1* and *FR-H2* loci and the effects of their expression. This model depicts a winter *vrn-H1* allele at *FR-H1/VRN-H1* and the Dicktoo-Nure allele at *FR-H2*, which harbors multiple copies of the *CBF4B–CBF2A* genomic region, boxed in red, green, and fuchsia. In the absence of *VRN-H1* expression *CBF*s are expressed to high levels at normal growth temperatures, exhibiting circadian rhythmicity and peaking towards the latter part of the day. CBF2 [135,159] (and likely also CBF4) activate expression of the HvCBF3/CBFIIId subgroup genes *CBF12*, *CBF15*, and *CBF16*. Increasing *CBF4B–CBF2A* genomic region copy numbers increases the pool of CBF12, CBF15, and CBF16. Light has a stimulatory effect upon *CBF12* transcripts and may similarly affect *CBF15* and *CBF16*. Based on recent findings in Arabidopsis, the CBFs are hypothesized to play a role in activating *PIF*-like genes in the barley genome, causing growth and developmental changes associated with the prostrate growth habit and leaf length reduction. Such activity may be a specialized role of the HvCBF3/CBFIIId subgroup genes. Expression of the *CBF*s becomes repressed through direct binding of the MADS-box protein VRN-H1 to the *CBF2*, *CBF4* and *CBF9* genomic regions. This may occur in conjunction with an as yet unidentified MADS-box protein that forms a heterodimer with VRN-H1, a mechanism typical of MADS box proteins. A spring *Vrn-H1* allele at *VRN-H1* results in constitutive expression of *VRN-H1* and the constitutive repression of *FR-H2*. The winter *vrn-H1* allele restricts *VRN-H1* transcript accumulation until other endogenous signals or external cues trigger its accumulation, the latter of which can be prolonged exposure to cold, LD photoperiod, or both, depending on the genotype. VRN-H2 represses *VRN-H1* but expression of *VRN-H2* requires LD; under SD the repressive role of VRN-H2 over *VRN-H1* is diminished. Facultative types like ‘Dicktoo’ and 88AB536-B lack a functional copy of the *VRN-H2* gene. ‘Dicktoo’ gates expression of *VRN-H1* solely through daylength—a minimum 12 h photoperiod is required for expression of *VRN-H1* in ‘Dicktoo’. The mechanism of control over *VRN-H1* expression in 88AB536-B is not defined and appears to be a combination of developmental and external cues. The top-down ordering of the *CBF* genes uses that of the physical map produced by Mareri et al. [137]. Single line spacing is used for genes present in clusters, double line spacing is used to denote separation by larger physical distances. *CBF*s in the HvCBF4/CBFIVa-d subgroup are indicated by blue text. Those in the HvCBF3/CBFIIId subgroup are indicated by black text. Gray background is used for *CBF*s showing expression while those that have been recalcitrant to detection are highlighted in yellow.

Some of what we know about *FR-H2* and its relationship to *VRN-H1* is illustrated in Figure 7. Yet much remains to be learned about this key locus. In the remainder of this review, I attempt to bridge aspects of what we have learned about the CBFs in the last decade or so and offer ideas as to how we might further our insight of winter-hardiness at the mechanistic level to be able to better predict it in breeding populations.

## 2. Winter-Hardiness and the Connection of the CBFs to the GA-GID1-DELLA Module

### 2.1. Morphological Traits Associated with Winter-Hardiness

About seven years ago, Blake Cooper, the senior wheat breeder at Limagrain for the Northern U.S. Plains, who was previously a breeder of malting barley at Busch Agricultural Resources, asked me whether I had characterized a particular barley line for *CBF* copy numbers after I described to him what we were finding regarding *CBF* copy number variation at the *FR-H2* locus. His comment about this particular line was that “it ALWAYS was [the] best two-row for winter survival... but it was also very tall, later than sin and did not yield well or malt at all…”

The theme of height and winter-hardiness are often noted to go together for cereals of the Triticeae. In the more extreme cold winter environments of North America, it has long been noted that the most winter-hardy wheats are tall and lodging-prone. Long-term variety testing trials of winter wheat show a high correlation between winter-hardiness and plant height and that this correlation is higher than the correlation between lodging and height [161,162].

Thomas and Schaalje [163] observed that when six winter wheats differing in their winter survival ability were planted as a composite of equal numbers, the two winter-hardy cultivars greatly increased in numbers relative to the less hardy cultivars following a severe winter. But the same two winter-hardy cultivars also increased relative to the less hardy cultivars following less severe winters. They also observed that the two winter-hardy cultivars had a greater interplant competitiveness over the other lines and speculated that that was due to the combined effect of the propensity for height with the ability to enter a prostrate growth habit, a trait long associated with the more winter-hardy wheats [164].

The prostrate, or rosette growth habit in wheat was initially thought to be strictly a cold induced phenomenon, but Roberts [165] showed that light was critical in inducing the plants to enter the prostrate growth habit. Reducing the amount of sunlight reaching plants growing in the field reduced the degree to which plants exhibited a prostrate habit [165,166]. To reproduce this phenotype in growth chambers a minimum total light energy of 350 klux (~5400 μM m^−2^s^−1^) per 24 h period was required; when grown under subthreshold illumination levels all genotypes exhibited an erect vertical phenotype [165,166]. This minimum can be achieved through high intensity under shorter periods or lower intensity under longer periods and increasing the light intensity beyond the critical threshold increased the degree to which plants exhibited the prostrate growth phenotype [165,166]. Thus, light acts in a quantitative manner on this trait since different light intensity–photoperiod combinations achieve the threshold for producing the rosette effect.

Roberts also noted that the cold hardiness of a wheat variety was highly correlated with the length of its leaves under field conditions, and that the greater the winter-hardiness of the variety, the greater the shortening of the leaves during the autumn period following planting [167]. Genetic analysis revealed that the cold hardiness and leaf length phenotypes were controlled by a locus on chromosome 5A that was linked to, but distinct from the locus controlling growth habit (winter vs. spring) and the rosette phenotype [93]. As the amount of light reaching the plants was reduced on field grown plants, leaf length increased but this phenotype was not reproduced in the growth chamber experiments [165]. Roberts suggested that the inability to reproduce the leaf length changes in the growth chambers was due to the growth chamber conditions in which plants were grown under a constant 3 °C, while the field grown plants did not experience 3 °C until late October [165]. Subsequent testing under different light intensity and temperature combinations indicated that a compact plant morphology (short leaves) could be obtained at normal growth temperatures using very high light intensity [168].

Assessing copy numbers of the HvCBF4/CBFIVa-d subgroup CBF genes in the chromosome substitution lines used by Roberts in the genetic analyses of leaf length indicated that *CBF* copies are much higher in the tall genotypes producing the greater shortening of leaf length under field conditions than in the lines exhibiting the less pronounced phenotype [169]. This data in conjunction with more recent experimental data from Arabidopsis indicating CBFs play a role in normal growth and development raises the question whether the CBFs play a role in the leaf length shortening, and possibly even height in the cereals. While it is speculation that the locus Roberts identified is *FR-H2*, the experimental data are consistent with the two being one in the same.

### 2.2. CBFs Play a Role in Normal Growth and Development

Recently Zhu and colleagues and Yang and colleagues used CRISPR/Cas9 to generate lines having null alleles for the three tandemly linked *Arabidopsis thaliana* chromosome 4 *CBF* genes [170,171]. The triple mutants exhibit lower germination rates, reduced root growth, and reduced shoot growth at normal growth temperatures [170,171]. But at 4 °C these differences in root growth did not occur [171].

Dong et al. [172] also observed reduced hypocotyl length in a line deleted for the *Arabidopsis CBF1* gene, and an elongated hypocotyl phenotype in *CBF1*, *CBF2*, and *CBF3* overexpressors under continuous (24 h) light. However, this phenotype was not observed in plants grown in continuous dark [172]. Identifying the *Phytochrome-Interacting Factor 4* (*PIF4*) and *PIF5* among a set of upregulated genes in the *CBF* overexpressors grown under continuous light, they then showed that CBF1 bound to CRT/DRE motifs in the upstream region of these two *PIF* genes using myc-tagged *CBF1* in chromatin immunoprecipitation experiments [172]. PIFs are DNA binding transcription factors associated with skotomorphogenesis, or the etiolated phenotype that occurs when plants are grown in the dark [173,174]. They are noted to activate expression of their target genes in the dark period and then diminish in abundance during the light period [173,174]. Notably however, the pronounced CBF1-mediated enhancements of hypocotyl elongation and PIF4 and PIF5 protein abundance that occurred at normal growth temperatures was abrogated at 4 °C [172].

In earlier studies Lee and Thomashow [175] showed a connection between the *Arabidopsis* CBFs and the PIFs at normal growth temperatures. In plants grown under a short-day photoperiod (8 h light and 16 h dark) and at normal growth temperatures, expression of the *Arabidopsis CBF*s and levels of freezing tolerance are significantly higher than in plants grown under LD photoperiod conditions (16 h light and 8 h dark) [175]. This photoperiodic difference was abolished in *pif4 pif7* double mutant lines, i.e., *CBF* levels and freezing tolerance of the *pif4 pif7* double mutants grown under LD was at the levels observed in the SD grown plants [175]. This PIF4 PIF7-mediated regulation of *CBF* expression occurs in association with the PIF4 PIF7 partner, the phytochrome B (PHYB) photoreceptor, through direct binding of the PIFs to the G and E box motifs in the upstream region of the *CBF* genes. Under the LD growth conditions PIF4 and PIF7 are at higher levels both at the transcript and protein levels, and in conjunction with PHYB, act to repress *CBF* expression and its pathway [175].

In other experiments Jiang et al. [176] also found that transcript levels of the *CBF*s were reduced in *PIF3* overexpressors grown under LD. ChIP experiments with a myc-tagged *PIF3* construct indicated that *PIF3* bound to the upstream regions of all three *CBF* genes [176]. The *pif3* loss of function mutants expressed the CBFs to much higher levels and showed increased freezing tolerance, providing further support for a connection between the CBF pathway and the PIFs [176].

Taken together these data suggest a feedback regulation between the CBF and PIFs that at first appears antagonistic. Under SD the CBFs are promoting expression of the PIFs, but under LD the PIFs act to repress the CBFs. Lee and Thomashow [175] suggest the circadian clock offsets the peak time of expression for *CBF* relative to that of *PIF4* and *PIF7* and that this may account for this apparent disparity. Additional light on the connection between the CBFs and the PIFs comes from more recent data from Jiang et al. [177], who show that the CBF1 and PIF3 proteins physically interact, and that this interaction likely plays a role in preventing the degradation of PIF3. In lines lacking functional copies of the *CBF*s, PIF3 levels decrease at 4 °C, whereas PIF1, PIF4, and PIF5 levels increase [177]. A model is presented in which the CBF-mediated stabilization of PIF3 enables it and the latter’s associated partner, PHYB, to remain active at normal temperatures in the light, which in turn leads to the degradation of PIF1, PIF4, and PIF5 [177]. This CBF-PIF data tells us that the CBFs are directing growth-promoting transcription factors in a light and temperature dependent manner.

In barley, peak *CBF* transcript levels also occur 6–8 h after subjective daybreak under SD [135]. Under LD peak levels are shifted about 4 h later in the subjective day, but with no major difference detected in the magnitude of levels between SD and LD [135]. Similarly, in wheat grown under LD, peak *CBF* expression occurred 8–14 h after subjective daybreak [132]. The expectation might then be that under natural daily light cycles in the field environment *CBF* transcript levels shift slightly earlier each day as days grow shorter, staying constant relative to the dark period. This pattern would also be expected to be reversed in the spring.

In carrying out the barley circadian experiments, light appeared to have a substantive affect upon the transcript levels of *CBF12*, a HvCBF3/CBFIIId subgroup gene. Much higher *CBF12* levels were detected under LD relative to SD, but when the SD-grown plants were transferred to 24 h light conditions, *CBF12* levels increased notably and remained high, whereas transferring the 16 h grown plants to constant dark, *CBF12* levels declined substantially. This affect did not occur for the HvCBF4/CBFIVa-d subgroup genes and was far more pronounced in the winter variety ‘Nure’, than in the spring variety ‘Trèmois’, suggesting the effect of light is mediated through CBF2 and CBF4 and their copy numbers [135]. More recent studies by Galiba and colleagues [178] indicate that *CBF14* transcript levels are enhanced by far-red light, a result supporting a connection between the CBFs, the growth controlling PIFs, and PHYB in the cereals.

### 2.3. The Semi-Dwarfing Wheat Rht-1 Genes, and Their Limitations in Stress Environments

The *reduced height* (*Rht-1*) semi-dwarfing genes, also referred to as the ‘Green Revolution’ genes encode DELLA proteins [179,180]. The *Rht-1* alleles conferring shorter stature are dominant over the wild type allele and have stop codon mutations in the *N*-terminal DELLA domain, which is the region of the protein that makes it responsive to the GA-mediated degradation pathway. In hexaploid bread wheat, functional *Rht-1* gene homoeologs exist for the AA, BB, and DD genomes on the respective group 4 homoeologous chromosomes. Dwarfing mutant alleles on the BB or the DD genome homoeologs are used in agriculture; dwarfing alleles for the AA genome have not yet been reported [180]. A single DELLA exists in barley, identified as *slender Slender1* [181]. However, it is not used in cultivated barley.

In the case of the wheat *Rht-B1b* and *Rht-D1b* semi-dwarf alleles, it is hypothesized (and remains to be shown) that a downstream MET codon enables translation of a shorter protein lacking the *N*-terminal DELLA domain, which because it lacks the DELLA domain, is not subject to degradation through the normal GA pathway [179,182]. This *N*-terminally truncated DELLA protein constitutively sequesters its target proteins, which includes the PIF3 DNA binding transcription factor. When bound by DELLA, PIF3 is unable to bind to target DNA sites [183]. Because the growth promoting PIF transcription factors are prevented from activating their target genes, growth is reduced; hence so is height and lodging.

Because DELLA proteins are targeted for degradation through GA signaling, when GA levels increase, DELLA levels decrease, and when GA levels decrease, DELLA levels increase. This occurs through a mechanism involving GIBBERELLIN INSENSITIVE DWARF1 (GID1), which undergoes a conformational change upon binding to bioactive GA, enabling the GA-activated GID1 isomer to bind to the DELLA protein leading to DELLA protein degradation through ubiquitination and subsequent proteosome-mediated degradation steps [182]. One environmental factor that acts to decrease GA levels is light and it does so through decreasing expression of genes affecting GA biosynthesis (*GA20ox*, *GA3ox*) and activating expression of genes in GA biodegradation (*GA2ox*). Plants are also more responsive to GA during the evening and recent studies by Nohales and Kay [184] show that one aspect of this regulation may occur through a mechanism in which the GID1 binding site of the DELLA protein is blocked by the GIGANTEA (GI) protein during the day. GI is degraded during the evening, in turn resulting in the degradation of DELLAs, which allows GA responsive genes to function. The regulation of genes affecting GA biosynthesis, catabolism, and sensitivity are also timed around the circadian clock such that GA activity is greater in the nighttime period [185,186,187]. DELLA levels are also reflective of total light dose received in a 24 h period [188]. In essence light level can be transmitted in a rheostat like manner to effect genes acting in light-driven development [188].

The ability of the *Rht-1* gene alleles to decrease height and the association between reduced height and a reduced incidence of lodging, has led to the incorporation of the *Rht-1* gene alleles into the germplasm of wheat breeding programs around the world. However there have been several instances in which the *Rht-1* genes have shown limitations. One of these is winter-hardiness, particularly in the case of winter wheats grown on the prairie provinces of Western Canada. And while assessments of winter survival in the offspring from crosses between a set of *Rht-1* semi-dwarfs having low to moderate winter-hardiness and a set of standard-height lines having very high winter-hardiness suggested obtaining winter-hardy semi-dwarfs was possible, obtaining *Rht-1* semi-dwarf cultivars possessing the same level of winter-hardiness as the original landraces has not been realized [56,161,162,189,190].

A second environmental situation in which the *Rht-1* semi-dwarfs are noted to underperform is in drought-prone environments. Jatayev et al. [191] list several studies in which the standard height cultivars and landraces outperform the semi-dwarfs in drought-prone environments. In the low rainfall western parts of the U.S. where dry land farming is practiced, the standard height cultivars are preferred because their coleoptiles emerge faster and the seedlings establish better in these dry soils than the *Rht-1* semi-dwarfs [192]. The standard height cultivars also often outperform the *Rht-1* semi-dwarfs across environments. Using the measures of performance that include yield, kernel weight, and test weight, Butler et al. [193] showed that the standard height recombinants outperformed the semi-dwarfs under both drought-prone and well-watered environments using a population of 140 recombinant inbred lines segregating for *Rht-B1b* and *Rht-D1b*. Nonetheless, the semi-dwarf alleles are increasingly present in most modern winter wheat grown across the U.S. [194].

To assess how the different *Rht-1* alleles might affect emergence in response to temperature, Pereira et al. [195] measured coleoptile length at progressively colder temperatures in isolines in which *Rht-B1b*, *Rht-D1b*, or both, were introgressed using a set of isolines created in a spring background and another set created in a winter background. At 18 °C, the standard height cultivars exhibited the greatest coleoptile length, isolines carrying reduced height alleles at both homoeologous loci exhibited the least, and those carrying a single *Rht-1* homoeoallele exhibited intermediate length. At progressively colder temperatures coleoptile length increased for all lines, but the differences conferred by the different *Rht-1* alleles become less apparent, and length was even greater in isolines having the *Rht-1* BB homeolog. Testing how GA might affect this response they also carried out these assays using four different concentrations of GA. At 18 °C, GA enhanced hypocotyl elongation in only the wild type line, but at 2 °C and 10 °C, GA enhanced hypocotyl elongation in all lines [195]. While this responsiveness was observed in both the winter and spring isolines, it was more pronounced in the spring isolines than in the winter isolines [195]. A conclusion drawn by Pereira et al. [195] is that the *Rht-B1b* and *Rht-D1b* mutant alleles were not responsive to GA at warmer temperatures but were responsive at colder temperatures. This at least in part provides an explanation for the significantly reduced emergence for lines carrying the *Rht-B1b* and *Rht-D1b* gene alleles following planting. Because the isolines carrying the *Rht-1* semi-dwarfing alleles are GA-responsive at the colder temperatures, this data could also be an indication that the *N-*terminal truncated DELLA protein is less efficiently translated at colder temperatures. In principle then, at colder temperatures the normal GA responsive mechanisms should be in place as a consequence of a functional copy of the *Rht-1-AA* homoeoallele and a functional copy of the BB or DD homoeoallele, depending upon which *Rht-1* semi dwarfing homoeoallele is incorporated.

### 2.4. Semi-Dwarfing Genes Used in Malting Barley and Their Limitations

In barley while mutant alleles for the DELLA protein-encoding gene *slender* are not used in cultivated germplasm, other loci effecting a semi-dwarfing habit have been used in the breeding of spring malting barley varieties, including *denso*, *ert-k*^32^, *ari-e* [196]. An allele of *denso*, *sdw1*, has been used to reduce height (and by association, confer lodging resistance) in spring feed type barley varieties, but it has not been used in malting barley [197,198]. Lines carrying the mutant alleles at *denso* and *ert-k*^32^ are responsive to GA, while the mutant alleles at *ari-e* are weakly responsive [199].

The *denso* allele has had the greatest contribution to malting barley, which was created in the Czech cultivar ‘Valticky’ and led to the cultivar ‘Diamant’ [196,200,201]. The *denso* allele from Diamant confers shorter height, increased tillering, and increased grain yield. However, it also imparts several “negative” traits including late flowering, lower kernel weight, low plumpness, and higher β-glucan content. Despite the negative attributes this allele is incorporated into many spring malting barley cultivars now grown across Europe and Great Britain [202]. However, it has not been used in the U.S. where it was also found to impart inferior yield, late flowering, low kernel weight, low kernel plumpness, and high β-glucan content [197,198,203]. *Ert-k32* and *ari-e* have had more limited use and neither appear to have been used in the U.S. in winter or spring barley.

Of the three loci, only the gene at the *sdw1*/*denso* locus has been identified at the molecular level, and characterization of its alleles provides an explanation as to why the *sdw1* allele has not been used in malting barley. Li and colleagues [204] used a candidate gene map-based approach, identifying *HvGA20ox2*, encoding the gibberellic acid (GA)-20 oxidase enzyme, which is involved in the GA biosynthetic pathway. Expression analysis revealed that the gene was expressed at four-fold lower in lines with *sdw1.d* (*denso*) relative to that of the *Sdw1* wt allele, and 60-fold lower in lines carrying the *sdw1.a* (*sdw1*) allele [205]. The *sdw1.a* allele is a null allele as the entire gene is deleted, and thus the expectation is that GA levels are far too low during the malting process [206]. In comparison, the *sdw1.d* is a seven base pair deletion in Exon 1, which results in a frame shift of the protein. Whether the frameshift in the *sdw1.d* Exon 1 allele renders the protein product nonfunctional is also unclear [206]. Transcription of the gene occurs, and it is suggested that a methionine codon downstream of the initiator codon may function to enable translation of a protein product that lacks the NH_3_-terminus, which is still able to carry out some activity [206]. However, two additional GA20ox genes exist in the barley genome, *HvGA20ox1* and *HvGA20ox3*, which are also involved in the GA biosynthetic pathway and both genes are expressed to higher levels in the mutants; *HvGA20ox1* more so in the *sdw1.a* background and *HvGA20ox3* more so in the *sdw1.d* background [206]. As such, one possibility the authors consider is that the increased expression of one of the other *GA20ox* genes compensates for the reduced activity—or loss—of the *HvGA20ox2* gene product in the *sdw1.d* mutants during the germination process in malting [206].

### 2.5. The Connection between Winter-Hardiness and Gibberellic Acid in the Cereals

Following discoveries that gibberellins have a stimulating effect on growth and development, and that plant extracts have gibberellins, there was interest in testing how winter-hardiness was affected by GA [207]. The earliest experiments entailed soaking seeds of winter-hardy wheat ‘Kharkov 22MC’ in a gibberellic acid solution prior to sowing of seed in the field in autumn. These experiments indicated winter survival was significantly reduced in plants grown from GA-treated seed. Other experiments tested the effects of the growth inhibitor 2-chloroethyltrimethylammonium chloride (chlorcholinchloride, CCC) and found that it led to earlier progression into a phenotype characteristic of winter-hardiness [208]. Subsequent growth chamber experiments confirmed freezing tolerance was enhanced by 2-chloroethyltrimethylammonium chloride and was reduced by GA [209,210]. It was also noted that the freeze damage phenotype of the growth chamber-grown CCC-treated plants was similar to the phenotype of plants naturally cold-hardened in autumn, while that of the GA-treated plants was more like that of winter wheat in spring [209,210]. The phenotypes observed by these authors are consistent with 2-chloroethyltrimethylammonium chloride restricting processes in the reproductive transition and GA promoting it.

### 2.6. The Connection between the CBF Pathway and Gibberellic Acid in Arabidopsis

About half a century after these studies a connection between the GA and CBF pathways was observed. *CBF* overexpressing plants, in addition to inducing freezing tolerance at normal growth temperatures, can exhibit severely stunted growth and delayed flowering phenotypes [118,211]. Noting that the shorter internode length phenotype of tomato plants overexpressing *Arabidopsis* *CBF1* was reminiscent of a GA deficiency, the application of GA_3_ to these overexpressors was found to restore height to normal, and so these authors proposed CBF1 might affect genes in hormone growth pathways [212]. Subsequent isolation of the activation-tagged *DDF1* line rescuable by GA_3_ supported the connection between the GA pathway and the CBFs [127].

*DREB1F*/*DDF1* overexpressing plants have significantly reduced levels of bioactive GA, as do *CBF1* overexpressing plants [127,213,214]. In these overexpressing plants, transcript levels of genes involved in GA biosynthesis are decreased (GA20-oxidases and GA3-oxidases) and the GA-deactivating GA 2-oxidase enzymes are increased, particularly those encoding *GA2ox3*, *GA2ox6*, *GA2ox7*, and *RGL3*; the latter being one of the five DELLA protein-encoding genes in *Arabidopsis*. In barley plants overexpressing *CBF15* the transcript levels of *GA2ox5*, a gene affecting GA catabolism are also substantially increased [160]. In the case of *GA2ox7* in *Arabidopsis*, DREB1F/DDF1 directly binds to CRT/DRE sites in its promoter [214]. The negative effects of *CBF1*/*DREB1F*/*DDF1* overexpression can be alleviated by compensatory mutations including a *GA2ox7* loss-of-function mutant that reduces expression of genes encoding these GA-deactivating enzymes, or DELLA loss of function mutants [213,214,215]. Notably, these DELLA mutants are also less freezing tolerant than the WT plants [213].

Recently, Lantzouni et al. [216] carried out experiments to identify the genes affected by GA and did this so as to identify those affected at normal growth temperatures (21 °C) and at cold temperatures (4 °C). These GA-temperature experiments revealed that very different gene sets are affected at the two temperatures, leading to the conclusion that the gene sets regulated by GA were specific for each of the two temperatures [216]. They also identified, via yeast two hybrid assays, about 260 transcription factors interacting directly with DELLA proteins. A subset of 14 of those specifically show responsiveness to GA at 4 °C. Within the latter set of 14 are several members of the GROWTH-REGULATING FACTOR family, a group of proteins that affect a diverse array of growth and developmental processes in plants, and in environmentally-responsive manners [217,218]. One of which, GRF5, suppressed *CBF* expression at cold temperatures when overexpressed [216]. Based on the finding that DELLAs interact with GRF5, and that GA acts to attenuate GRF5, Lantzouni et al. [216] suggest DELLAs act as coactivators of *CBF* expression. In light of the role the CBFs may be playing in growth and development at normal temperatures, it also seems likely that many of those DELLA-interacting transcription factors responding to GA at normal growth temperatures also overlap with those regulated by the CBFs at normal growth temperatures.

## 3. Future Directions

### Understanding Winter-Hardiness and Predicting It in Future Breeding Populations

Given that *FR-H2* is repeatedly identified as the primary locus affecting low temperature tolerance [88,89,90], and that *CBF2A–CBF4B* genomic region copy numbers positively correlate with low temperature tolerance [100,135,136], it seems important to incorporate these alleles into modern winter malting barley lines. A reliable breeder-friendly molecular approach, ideal to assess copy numbers needs to be developed. DNA blot hybridization and qPCR are too labor intensive for assessing numbers of lines comprising breeding populations. If haplotype associations between alleles on a high throughput genotyping platform such as the Barley 50k iSelect SNP Array reliably predict copy number, then this genotyping platform or markers extracted from it could be used as a proxy [219].

It may also be important to have an idea of the stability of the genomic region in segregating populations. The high copy number alleles show they are stable in an inbred line being selfed, but non-parental recombinants have been observed in several lines resulting from crosses between individuals having different copy number including the complete deletion of the *CBF2A–CBF4A* gene cluster from a high copy number allele, suggesting nonallelic homologous recombination occurred [135]. A clear example of nonallelic homologous recombination is the barley ‘Trèmois’ *CBF2B-A* allele, in which an in-frame fusion occurred between the coding sequences of the *CBF2B* and *CBF2A* paralogs [100]. A similar in-frame fusion occurred in the tandem cluster of CBFs in tomato between adjacent genes [220]. Determining the structure of the non-parental alleles in barley and the distinctly different winter *FR-H2* alleles such as those present in ‘Kompolti korai’ and ‘Mumie Pori’ may also reveal recombination hot spots and potentially even lead to strategies to induce non-allelic homologous recombination at will to generate high copy number alleles.

Fundamental questions are whether the *FR-H2* CBFs play a role in plant establishment in the autumn and are they configuring a developmental program that enables the plant to survive long term subzero temperatures, how and when is their expression altered over the course of development in the field, and how do *CBF2A*–*CBF4B* genomic region copy numbers play into this. Do copy numbers confer measurable, incremental levels of endurance for growth under prolonged cold and freezing conditions? Or are they simply increasing throughput through the genetic program during the vegetative growth phase? Quite simply does doubling the dose of *CBF2* and *CBF4* double the dose of the CBF regulon? A related and more specific question is whether the *CBF*s affect root formation, in the autumn during establishment and following freezing conditions that injure or kill roots.

Expression of the *CBF*s are deactivated by the reproductive transition but it is not clear exactly when this occurs and whether this is an abrupt halting across all cells and tissues, or whether their expression gradually diminishes over time as *VRN-H1* levels increase. If they indeed play a role in root growth or root morphogenesis, then it seems critical that they still be actively expressed at least through spring green up in those cells required for root morphogenesis. In ‘Dicktoo’, cessation of activated expression is predicted to occur at or around the time of the vernal equinox (about 20 March). Thus, recapitulating the ‘Dicktoo’ photoperiod phenotype in malting barley lines could be one of the simplest means to breed winter-hardy malting barley. But for true winter genotypes, there will be a steady increase of *VRN-H1* under the short days of winter, and this could be highly variable across genotypes and environments, depending on the particular allelic combination those genotypes possesses and how those haplotypes respond to the environment. Knowing the transition point at which *VRN-H1* levels reach some critical threshold and *CBF*s are no longer actively expressed, and in turn being able to predict that for other genotypes based on their haplotype state would enable breeding for winter-hardiness through the ability to predict when that transition would occur with a given haplotype state.

Returning to the Top 10 winter-hardy lines in Table 1, there are multiple ways to skin the winter-hardiness cat. ‘Dicktoo’ (#2) achieves winter-hardiness in part through photoperiod control over *VRN-H1* and a winter allele at *FR-H2*. ‘Kearney’ (#1) has not been characterized at the molecular level, but its phenotypic characterization by the NPGS indicates that it is a winter type mixed with facultative types. Thus, it too could be through a photoperiod control over *VRN-H1* and a winter allele at *FR-H2*. NE 62434 (#5) likely possesses the Mumie Pori *FR-H2* allele, as it is a single plant selection from the Korean landrace Cha-Dae-Maec. This *FR-H2* allele is distinct from that of the Dicktoo-Nure allele in multiple ways, most notable of which appears to be the presence of multiple copies of *CBF14* [135]. As for material from Northeast Asia [158], Cha-Dae-Maec and its derivative NE 62434 (#5) may also carry alleles enhancing the winter growth habit. Thus, obtaining a physical map of the Mumie Pori *FR-H2* winter allele and determining that transition point at which the *CBF* genes of Mumie Pori become deactivated also seem highly meritorious pursuits for understanding and being able to predict winter-hardiness.

Another of the top 10 lines for which we have some mechanistic insight is ‘Admire’ (#7). This line possesses 7–8 copies of the *CBF2A–CBF4B* genomic region and expresses these two genes and several of the other *FR-H2 CBF*s to much higher levels than winter barley lines possessing the 2–3 copy number Dicktoo-Nure allele [76,135]. Characterization of this phenotype indicates that the increased copy numbers of *CBF2* and *CBF4* lead to higher transcript levels of the other *CBF*s through binding to target sites in their genomic regions and activating their expression—at least those possessing target sites. Still there may be other mechanisms that are not yet clear as *CBF14* exhibited higher transcript levels in a set of lines possessing higher copy numbers of the *CBF2A–CBF4B* genomic region related by descent to ‘Admire’. However no CRT/DRE motifs were identified in its upstream region, it did not exhibit increased levels in *CBF2* overexpressors, and it does not appear to be present in increased copy numbers in the Admire variant of the Dicktoo-Nure allele [135]. One possible explanation for the higher transcript level of *CBF14* for the Admire variant is that the structural environment is affecting expression of neighboring genes outside the variable unit, a phenomenon that occurs with structural variants in the human genome [221,222].

The insight with the different *FR-H2* winter allelic variants and their expression also brings us back to a fundamental question of why so many CBFs. The gene family at *FR-H2* is substantial in size, both in terms of coding sequence diversity and the copy numbers of individual paralogs. Do each of the genes carry out specific functions or is coding sequence diversity and copy numbers simply a means to maximize expression levels? Creating mutant lines for one or more of the *FR-H2 CBF*s would be very informative but it could be confounded by redundancy. More fruitful may be to first assess the role the copy number variation plays on growth and developmental process and the transcriptome, and how these are affected by the environment. For example, the observation that light seems to have a strong promotive effect on levels of *CBF12* (and possibly that of other HvCBF3/CBFIIId subgroup CBFs) and that this appears to be reflective of *CBF2* and *CBF4* levels, suggests a more complex positive feedback regulatory control mechanism is at work. Other questions of fundamental importance relate to regulatory control over expression of the *CBF*s, the pathways they control and how they are established in normal growth and development in the field. Having a clearer picture of the mechanisms determining the deactivation of the *CBF*s and the growth phase transition state would be extremely valuable for breeders to predict winter-hardiness.

Determining the transcriptome of plants over the normal course of development and doing this with set of lines differing in *CBF2A*–*CBF4B* genomic region copy numbers could provide insight into the role these allelic variants play in affecting target gene sets and the phenotypes associated with winter-hardiness and affected in the field environment. The trait phenotyping experiments could be carried out using biparental populations derived from lines differing in copy number, while sets of reciprocal NILs could be used for assessing the effects copy number has on the transcriptome. Combining this approach with one utilizing CBF overexpressing lines in a winter barley background could aid efforts to identify the barley CBF regulon. These lines would also aid in identifying how environmental factors impinge upon those developmental programs.

Identifying the transcriptome affected by the *FR-H2* CBFs and assessing how this transcriptome is affected in the wheat *Rht-1* and barley *Slender1* mutant backgrounds also merits attention because it is expected to provide mechanistic insight into CBF-mediated growth processes, and the restrictions the *Rht-1* semi-dwarfs may be placing on performance, particularly in the extreme cold and drought-prone environments. Given the climate change models put before us, this pursuit also seems highly meritorious. The coleoptile elongation experiments carried out by Pereira et al. [195] that show the effect of the wheat semi-dwarfing *Rht-1* alleles is pronounced at normal temperatures and less so or nonexistent at colder temperatures, and the experiments of Zhu and colleagues and Yang and colleagues indicating the *CBF*s have a strong positive affect upon growth at normal growth temperatures but not at colder temperatures suggests a critical yet unexplored aspect of the “Freezing Tolerance” *CBF* genes lies not just with cold temperatures, but in the growth period leading up to cold temperatures. The sequestration of PIF3 like orthologs by truncated wheat *Rht-1* and barley *Slender1* proteins is expected to alter (presumably lessen) the affects the CBFs have upon growth at normal growth temperatures.

The connection of the CBFs to the GA-GID1-DELLA module also relates back to winter-hardiness and the findings of Roberts [93,165] and of Thomas and Schaalje [163]. That *CBF*s appear to have a positive effect upon genes affecting GA catabolism, and a negative effect upon those affecting GA biosynthesis and yet they are expressed at a time point that coincides with an expected greater activity of GA may seem like a conundrum, but this apparent conundrum may actually be a key component of the mechanism the tall winter-hardy wheats coopt, which we observe in the field as associations of winter-hardiness levels with greater leaf length shortening, degree of the prostrate growth habit, and interplant competitiveness. Thus, unravelling this conundrum would also advance our understanding of the mechanisms the Triticeae cereals are utilizing in setting the stage for winter survival.

Along this same line of thinking it must also be kept in mind that the genes expressed in the vegetative growth phase are likely to be altered by the transition to the reproductive phase. For example, in rice the expression of the GA catabolism gene *GA2ox1* significantly decreases in shoot apices once the plant transitions from the vegetative phase to the reproductive phase [223]. In wheat GA enhances spike development, and the genes involved in GA biosynthesis are expressed to much higher levels in the shoot apical meristem under growth conditions enhancing reproductive development, but expression of *VRN-1* is required for GA to have this affect [224]. In barley the genomic region encompassing the GA biosynthesis gene *GA20ox2* is bound by *VRN-H1* as are the genomic regions encompassing *CBF2*, *CBF4*, and *CBF9* [153]. However it seems likely that different factors are recruited to the *GA20ox2* genomic region than to the *CBF* genomic region as the *CBF*s are repressed by VRN-H1. Identifying allelic variants less responsive to GA following the reproductive transition that are still fully active in the vegetative phase may also provide a means to reduce height without compromising the CBF pathway during the vegetative phase.

While DELLA mutants are not used in barley, identifying the CBF transcriptome affected by the DELLA mutations in wheat is expected to reveal those genes functioning in conferring endurance for prolonged periods in extreme cold environments, and in turn where to look for allelic variants that might enhance performance under these conditions. The low survivability of offspring from winter × spring crosses tells us the inheritance of winter-hardiness is likely to be much more than two alleles at two loci, *FR-H1* (*VRN-H1*) and *FR-H2*. As one trend in the breeding of spring barley has been the selection of early maturing lines, allelic variation across the genome that enhances vegetative growth processes may have been inadvertently lost or even selected against in the breeding of spring barley.

## Figures and Tables

**Table 1:** Top 10 winter-hardy lines of 204 tested at 111 sites across North America 1937–1956.

Rank	Name	CIho No.	Pedigree
1	Kearney	7580	Selection from Composite Cross III (CIho 5530)
2	Dicktoo	5529	Selection #2 from unknown cross made pre 1917 at Dickinson, North Dakota
3	OAC * 4GH1	10,096	Kenate/Wong
4	MO B893	9516	Selection from Ludwig (CI 7525)
5	NE 62434	9581	Selection from Korean landrace Cha-Dae-Maec (PI 157656, CI 7404)
6	NE 53417	9580	Wong/Ludwig
7	Admire	6377	Selection from a Kansas farmer’s field
8	Kansas South Central	6376	Bulk seed from Kansas farmers’ fields
9	OAE 30GH10	10,097	Kenate/Wong
10	Purdue B466A7-7-7-2	10,102	Purdue 28156A3-2-2-2/Wisconsin H42-5-4-5-1-1/Kentucky1/ Purdue 400-17/Wong

* Ontario Agricultural College.

## Data Availability

Not applicable.
